# A review of mathematical modeling of bone remodeling from a systems biology perspective

**DOI:** 10.3389/fsysb.2024.1368555

**Published:** 2024-04-09

**Authors:** Carley V. Cook, Ariel M. Lighty, Brenda J. Smith, Ashlee N. Ford Versypt

**Affiliations:** ^1^ Department of Chemical and Biological Engineering, University at Buffalo, The State University of New York, Buffalo, NY, United States; ^2^ Indiana Center for Musculoskeletal Health, School of Medicine, Indiana University, Indianapolis, IN, United States; ^3^ Department of Obstetrics and Gynecology, School of Medicine, Indiana University, Indianapolis, IN, United States; ^4^ Department of Biomedical Engineering, University at Buffalo, The State University of New York, Buffalo, NY, United States; ^5^ Institute for Artificial Intelligence and Data Science, University at Buffalo, The State University of New York, Buffalo, NY, United States

**Keywords:** bone remodeling cycle, basic multicellular unit, bone chemical signaling, bone cells, osteoimmunology, mechanistic modeling, differential equations, agent-based modeling

## Abstract

Bone remodeling is an essential, delicately balanced physiological process of coordinated activity of bone cells that remove and deposit new bone tissue in the adult skeleton. Due to the complex nature of this process, many mathematical models of bone remodeling have been developed. Each of these models has unique features, but they have underlying patterns. In this review, the authors highlight the important aspects frequently found in mathematical models for bone remodeling and discuss how and why these aspects are included when considering the physiology of the bone basic multicellular unit, which is the term used for the collection of cells responsible for bone remodeling. The review also emphasizes the view of bone remodeling from a systems biology perspective. Understanding the systemic mechanisms involved in remodeling will help provide information on bone pathology associated with aging, endocrine disorders, cancers, and inflammatory conditions and enhance systems pharmacology. Furthermore, some features of the bone remodeling cycle and interactions with other organ systems that have not yet been modeled mathematically are discussed as promising future directions in the field.

## 1 Introduction

Bone is a dynamic living tissue that plays a crucial role in providing mechanical support to the body and maintaining systemic homeostasis. Bone remodeling is the delicately balanced process of coordinated activity of bone cells that remove and deposit new bone tissue to renew the adult skeleton (Allen and Burr, 2014; Bellido et al., 2014). Multiple biochemical, physical, and mechanical factors within the bone microenvironment and throughout the body regulate bone cell activity. When these factors operate within a homeostatic range, bone removal and formation activities of bone cells are balanced, and the bone remodeling cycle ends without a net change in bone volume or mass during tissue turnover. Perturbations outside this range can cause an imbalance between bone removal and formation leading to pathological bone loss (Allen and Burr, 2014). The pathologies of bone loss due to conditions such as renal failure, cancer, diabetes, and age-related bone loss differ (as well as their responses to treatment). The drive to understand bone pathologies and to design effective therapeutics leads researchers to study the local and systemic mechanisms that regulate bone remodeling. Developing mathematical models that could be adapted to the unique aspects of these scenarios would provide a powerful tool.

Mechanisms of bone remodeling are complex to capture in traditional *in vivo* and *in vitro* experiments due to the dynamic nature of the cell populations involved and the complexity of their local and systemic interactions. For preclinical *in vivo* studies, the measurements that can be performed at the tissue or mechanistic level are limited by the number of timepoints typically relegated to cross-sectional study designs. With *in vitro* studies, it is challenging to create an environment that allows the cells to respond to systemic changes that influence the *in vivo* bone microenvironment. Mathematical modeling captures the dynamics of cell populations and simulates complex interactions over time, integrating effects from multiple scales locally and systemically. It is widely used for understanding bone remodeling biology and hormone dynamics. By enhancing existing models from a systems biology perspective, we can bridge the gap between molecular signaling and clinically measurable properties that correspond to tissue and patient phenotypes. This perspective considers the human body as an integrated whole with multiple interacting systems, requiring the integration of diverse data sets to understand, design, and control therapeutic responses.

Many mathematical models have been developed to enhance the understanding of the bone remodeling process. Here, we review 88 such models, with annotations included in Supplementary Tables S1–S3. These models primarily fall into two categories for types of effects that they consider: biomechanical and biochemical. Biomechanical models aim to describe how the morphology, structural integrity, and mechanical loading of the bone matrix affect the evolution of bone (van Oers et al., 2008; Lerebours et al., 2016; Kameo et al., 2020; Calvo-Gallego et al., 2023). Some biomechanical models incorporate individual bone cell dynamics in a simplified manner. In contrast, biochemical models focus on a detailed representation of the biochemical processes governing bone cell populations. Biochemical models incorporate interactions between key molecular signals and bone cells but often neglect critical mechanical signals. Mechano-chemo-biological models are a newer third category of bone remodeling models to address the need for sufficient biomechanical and biochemical detail (Lerebours et al., 2016; Martin et al., 2019; Ashrafi et al., 2020; Ait Oumghar et al., 2020; Pant et al., 2021).

Here, we provide a comprehensive review of biochemical mathematical models of bone remodeling. We include a few mechano-chemo-biological models in this review to highlight how they consider changes to the biochemical bone remodeling network. Unique among other related reviews (Gerhard et al., 2009; Geris et al., 2009; Pivonka and Komarova, 2010; Webster and Müller, 2011; Riggs and Cremers, 2019; Coelho et al., 2020), our review analyzes the mathematical forms used to represent the physiological processes of bone remodeling, highlights important local and systemic biological features found in mathematical models, and synthesizes these into comprehensive tables that should be useful to others interested in building or adapting such models (Tables 1–7). Spatiotemporal biochemical models (van Oers et al., 2008; Ryser et al., 2009; Ayati et al., 2010; Ryser et al., 2010; Buenzli et al., 2011; Buenzli et al., 2012a; Graham and Ayati, 2012; Ryser et al., 2012; Araujo et al., 2014; Buenzli et al., 2014; Buenzli, 2015; Lerebours et al., 2016; Ryser and Murgas, 2017; Arias et al., 2018; Peyroteo et al., 2019; Kameo et al., 2020; Taylor-King et al., 2020; Baldonedo et al., 2021; Calvo-Gallego et al., 2023; Idrees and Sohail, 2023) are categorized in Table 1. Temporal biochemical models are organized based on which of two prevailing mathematical formulations are used to describe bone cell population dynamics and their biochemical signaling dynamics in the BMU. The temporal models that adopt the power law approach defined in Section 4.1 (Komarova et al., 2003; Komarova, 2005; Garzón-Alvarado, 2012; Liò et al., 2012; Graham et al., 2013; Jerez and Chen, 2015; Chen-Charpentier and Diakite, 2016; Coelho et al., 2016; Jerez et al., 2018; Camacho and Jerez, 2019; Idrees et al., 2019; Javed et al., 2019; Idrees and Sohail, 2020; Miranda et al., 2020; Camacho and Jerez, 2021; Islam et al., 2021; Cook et al., 2022) are categorized in Table 2, described in Section 5.2, and detailed in Supplementary Table S1. The temporal models that adopt the mass action kinetics approach defined in Section 4.2 (Lemaire et al., 2004; Marathe et al., 2008; Pivonka et al., 2008; Peterson and Riggs, 2010; Pivonka et al., 2010; Marathe et al., 2011; Schmidt et al., 2011; Wang et al., 2011; Buenzli et al., 2012b; Peterson and Riggs, 2012; Ross et al., 2012; Wang and Qin, 2012; Pivonka et al., 2013; Post et al., 2013; Scheiner et al., 2013; Ji et al., 2014; Scheiner et al., 2014; Berkhout et al., 2015; Eudy et al., 2015; Berkhout et al., 2016; Lee and Okos, 2016; Farhat et al., 2017; Ross et al., 2017; Hasegawa and Duffull, 2018; Pastrama et al., 2018; Ji et al., 2019; Lemaire and Cox, 2019; Martin et al., 2019; Martínez-Reina and Pivonka, 2019; Trichilo et al., 2019; Zhang and Mager, 2019; Ashrafi et al., 2020; Bahia et al., 2020; Ji et al., 2020; Lavaill et al., 2020; Martin et al., 2020; Larcher and Scheiner, 2021) are categorized in Table 3, described in Section 5.3, and detailed in Supplementary Table S2. The temporal models that do not explicitly use the power law or mass action kinetics approaches (Kroll, 2000; Rattanakul et al., 2003; Martin and Buckland-Wright, 2004; Moroz et al., 2006; Moroz and Wimpenny, 2007; Akchurin et al., 2008; Ji et al., 2012; Proctor and Gartland, 2016; Chaiya and Rattanakul, 2017; Javed et al., 2018; Zhao and Zhang, 2019; Javed et al., 2020; Nutini et al., 2021; Jorg et al., 2022) are categorized in Table 4 and detailed in Supplementary Table S3. A recent review of existing mechanical models of bone remodeling is provided by Della Corte et al. (2020), and Pant et al. (2021) reviews mechanical and mechano-chemo-biological and points to opportunities for integrating mechanics, biology, and biochemistry at the cellular and molecular scales. The review of Ait Oumghar et al. (2020) complements our review of biochemical models but distinctly emphasizes the experimental evidence for biochemical models of bone diseases, such as osteoporosis, Paget’s disease, and bone metastases. Ledoux et al. (2022) organizes their discussion of existing models by the biological features, but their focus is on summarizing a wealth of relevant clinical data for parameterizing such models. We intend for the present review to motivate systems biology researchers to look at bone beyond the local microenvironment to better understand the complexities of bone within the body as an integrated whole while still using past accomplishments in localized mathematical modeling and experimental data.

**TABLE 1:** Overview of cells and signaling molecules commonly included in spatiotemporal biochemical models of bone remodeling.

References	pOCL	OCL	pOBL	OBL	OCY	RANK	RANKL	OPG	A&P	TGFβ	PTH	Other
ABMs												
van Oers et al. (2008)*		x		x	x							
Buenzli et al. (2012a)		x										
Arias et al. (2018)	x	x	x	x	x							
Taylor-King et al. (2020)			x	x	x							
ABMs & PDEs												
Araujo et al. (2014)	x	x	x	x			x			x		
PDEs												
Ryser et al. (2009)		x		x			x	x	x			
Ayati et al. (2010)		x		x					x			
Ryser et al. (2010)		x		x			x	x	x			
Buenzli et al. (2011)		x	x	x		x	x	x		x	x	
Graham and Ayati (2012)		x		x					x			
Ryser et al. (2012)	x	x		x			x	x	x		x	
Buenzli et al. (2014)		x	x	x		x	x	x		x	x	
Buenzli (2015)				x	x							
Lerebours et al. (2016)*	x	x	x	x		x	x	x		x	x	
Ryser and Murgas (2017)		x		x	x				x			
Peyroteo et al. (2019)		x		x					x			
Kameo et al. (2020)*		x		x	x		x	x				SCL
Baldonedo et al. (2021)		x	x	x	x				x			SCL
Calvo-Gallego et al. (2023)*	x	x	x	x	x	x	x	x		x	x	
Idrees and Sohail (2023)		x		x					x		x	

The * symbol indicates models that include biomechanical features. The modeling approaches and additional details are available in Supplementary Tables S1–S3. Abbreviations: ABMs, agent-based models; PDEs, partial differential equations; pOCL, preosteoclasts; OCL, osteoclasts; pOBL, preosteoblasts; OBL, osteoblasts; OCY, osteocytes; RANK, receptor activator of nuclear factor kappa-B; RANKL, receptor activator of nuclear factor kappa-B ligand; OPG, osteoprotegerin; A&P, general autocrine and paracrine signaling; TGF-β, transforming growth factor beta; PTH, parathyroid hormone; SCL, sclerostin.

**TABLE 2:** Overview of cells and signaling molecules commonly included in ODEs-based temporal biochemical models of bone remodeling that follow the power law approach.

References	pOCL	OCL	pOBL	OBL	OCY	RANKL	TGFβ	SCL	Wnt	PTH
Komarova et al. (2003)		x		x						
Komarova (2005)		x		x						x
Garzón-Alvarado (2012)		x		x			x			x
Liò et al. (2012) ^†^		x		x		x				
Graham et al. (2013)		x	x	x	x			x		
Jerez and Chen (2015)		x		x						
Chen-Charpentier and Diakite (2016)		x		x						
Coelho et al. (2016)	x	x	x	x						x
Jerez et al. (2018) ^†^		x		x						
Camacho and Jerez (2019)		x		x						
Idrees et al. (2019)		x	x	x						x
Javed et al. (2019)		x		x		x				
Idrees and Sohail (2020)		x		x						
Miranda et al. (2020)		x		x						
Camacho and Jerez (2021)		x		x			x		x	
Islam et al. (2021)		x	x	x	x			x	x	
Cook et al. (2022)		x	x	x	x			x	x	

All models include general autocrine and paracrine (A&P) signaling. The ^†^ symbol indicates models that include stochasticity. Additional details are available in Supplementary Table S1. Abbreviations: ODEs, ordinary differential equations; pOCL, preosteoclasts; OCL, osteoclasts; pOBL, preosteoblasts; OBL, osteoblasts; OCY, osteocytes; RANKL, receptor activator of nuclear factor kappa-B ligand; TGF-β, transforming growth factor beta; SCL, sclerostin; Wnt, wingless-related integration site; PTH, parathyroid hormone.

**TABLE 3:** Overview of cells and signaling molecules commonly included in ODEs-based temporal biochemical models of bone remodeling that follow the mass action kinetics approach.

References	pOCL	OCL	pOBL	OBL	OCY	SCL	Wnt
Lemaire et al. (2004)		x	x	x			
Marathe et al. (2008)		x	x	x			
Pivonka et al. (2008)		x	x	x			
Peterson and Riggs (2010)		x	x	x			
Pivonka et al. (2010)		x	x	x			
Marathe et al. (2011)		x	x	x			
Schmidt et al. (2011)		x		x			
Wang et al. (2011)		x	x	x			
Buenzli et al. (2012b)		x	x	x			x
Peterson and Riggs (2012)		x	x	x			
Ross et al. (2012)		x	x	x			
Wang and Qin (2012)		x	x	x			
Pivonka et al. (2013)*	x	x	x	x			
Post et al. (2013)		x		x			
Scheiner et al. (2013)*		x	x	x			
Ji et al. (2014)		x	x	x			
Scheiner et al. (2014)*		x	x	x			
Berkhout et al. (2015)		x		x			
Eudy et al. (2015)		x	x	x	x		x
Berkhout et al. (2016)		x		x			
Lee and Okos (2016)		x	x	x			
Farhat et al. (2017)		x	x	x			x
Ross et al. (2017)		x	x	x			
Hasegawa and Duffull (2018)		x	x	x			
Pastrama et al. (2018)*		x	x	x			
Ji et al. (2019)		x	x	x			
Lemaire and Cox (2019)		x	x	x		x	x
Martin et al. (2019)*		x	x	x	x	x	x
Martínez-Reina and Pivonka (2019)*		x	x	x			
Trichilo et al. (2019)		x	x	x			
Zhang and Mager (2019)		x	x	x			
Ashrafi et al. (2020)*		x	x	x			
Bahia et al. (2020)*		x	x	x			
Ji et al. (2020)		x	x	x			
Lavaill et al. (2020)*		x	x	x			
Martin et al. (2020)*		x	x	x	x	x	x
Larcher and Scheiner (2021)*		x	x	x			

All models include RANK, RANKL, OPG, TGF-β, and PTH. The * symbol indicates models that include biomechanical features. Additional details are available in Supplementary Table S2. Abbreviations: ODEs, ordinary differential equations; pOCL, preosteoclasts; OCL, osteoclasts; pOBL, preosteoblasts; OBL, osteoblasts; OCY, osteocytes; RANK, receptor activator of nuclear factor kappa-B; RANKL, receptor activator of nuclear factor kappa-B ligand; OPG, osteoprotegerin; TGF-β, transforming growth factor beta; PTH, parathyroid hormone; SCL, sclerostin; Wnt, wingless-related integration site.

**TABLE 4:** Overview of cells and signaling molecules commonly included in ODEs-based temporal biochemical models of bone remodeling that do not follow the power law or mass action kinetics approaches.

References	pOCL	pOBL	OCY	A&P	RANK	RANKL	OPG	TGFβ	SCL	Wnt	PTH
Kroll (2000)	x	x									x
Rattanakul et al. (2003)					x	x					x
Martin and Buckland-Wright (2004)						x	x	x			
Moroz et al. (2006)*			x	x							
Moroz and Wimpenny (2007)*			x	x							
Akchurin et al. (2008)						x					
Ji et al. (2012)											
Proctor and Gartland (2016)*	x	x	x		x	x	x	x	x	x	x
Chaiya and Rattanakul (2017)											x
Javed et al. (2018)	x	x				x	x		x		
Zhao and Zhang (2019)											x
Javed et al. (2020)						x	x				
Nutini et al. (2021)*			x			x	x		x		
Jorg et al. (2022)	x	x							x		

All models include OCL and OBL except Martin and Buckland-Wright (2004) and Akchurin et al. (2008), which only include OCL, and Nutini et al. (2021), which only includes OBL. The * symbol indicates models that include biomechanical features. Additional details are available in Supplementary Table S3. Abbreviations: ODEs, ordinary differential equations; OCL, osteoclasts; OBL, osteoblasts; pOCL, preosteoclasts; pOBL, preosteoblasts; OCY, osteocytes; A&P, general autocrine and paracrine signaling; RANK, receptor activator of nuclear factor kappa-B; RANKL, receptor activator of nuclear factor kappa-B ligand; OPG, osteoprotegerin; TGF-β, transforming growth factor beta; SCL, sclerostin; Wnt, wingless-related integration site; PTH, parathyroid hormone.

**TABLE 5:** Representation of cytokines in mathematical models of bone remodeling.

References	Cytokine	Variable type	Cytokine interactions
Kroll (2000) ^3^, Idrees et al. (2019) ^1^	IL-6	Dynamic	Stimulates OCL formation (time-delayed)
			Production rate by OBL
			Elimination rate of IL-6
Wang et al. (2011) ^2^, Ji et al. (2014) ^2^, Ji et al. (2020) ^2^	IL-6	Dynamic	Production rate by BMSC via TGF-β
			Stimulates RANKL expression by pOBL
			Production by tumor-BMSC adhesion via VLA4
			Stimulates tumor cell proliferation
Martin and Buckland-Wright (2004) ^3,‡^	MCSF	Constant	Presence in healthy bone tissue
Pivonka et al. (2013) ^2^, Lerebours et al. (2016) ^2,*§*^	MCSF	Constant	Binding on uncommitted OCL
Proctor and Gartland (2016) ^3,‡^	MCSF	Dynamic	Stimulates HSC differentiation to pOCL
			Production by OBL progenitor
			Production by pOBL and OBL
			Production by PTH-stimulated pOBL and OBL
			Degradation rate of MCSF
Garzón-Alvarado (2012) ^1,‡^	IGF	Dynamic	Inhibits OBL differentiation
			Production by tumor cells
Lee and Okos (2016) ^2^	IGF-1	Dynamic	Binding kinetics to IGFBP3 receptor
			Stimulates pOBL formation
			Stimulates pOBL differentiation to OBL

The modeling approach is denoted by superscripts as follows: (1) power law, (2) mass action kinetics, or (3) neither. All models that follow the mass action kinetics approach include RANK, RANKL, OPG, and TGF-β. Models that do not follow this approach but include any of the signals above are indicated by the ^‡^ symbol. Spatiotemporal models are indicated by the ^*§*^ symbol. Abbreviations: RANK, receptor activator of nuclear factor kappa-B; RANKL, receptor activator of nuclear factor kappa-B ligand; OPG, osteoprotegerin; TGF-β, IL-6, transforming growth factor beta; interleukin-6; OCL, osteoclasts; OBL, osteoblasts; BMSC, bone marrow stromal cells also known as mesenchymal stem cells; pOBL, preosteoblasts; VLA4, very late antigen-4; MCSF, macrophage colony-stimulating factor; HSC, hematopoietic stem cells; pOCL, preosteoclasts; PTH, parathyroid hormone; IGF, insulin-like growth factor; IGFBP3, insulin-like growth factor binding protein 3.

**TABLE 6:** Representation of immune cells in mathematical models of bone remodeling.

References	Immune Cell(s)	Variable type	Cell interactions
Akchurin et al. (2008) ^3,‡^	Monocytes	Dynamic	Proliferation and fusion of monocytes
			Differentiation to OCL
Proctor and Gartland (2016) ^3,‡^	HSC	Constant	Differentiation to pOCL by MCSF
Islam et al. (2021) ^1^	Naïve CD4^+^ T cells, Tregs	Dynamic	Differentiation of Naïve T to Tregs
			Effects of butyrate on T cell differentiation
			Migration of Tregs between compartments
			Effects of Tregs on TGF-β fold change

The modeling approach is denoted by superscripts as follows: (1) power law, (2) mass action kinetics, or (3) neither. All models that follow the mass action kinetics approach include RANK, RANKL, OPG, and TGF-β. Models that do not follow this approach but include any of the signals above are indicated by the ^‡^ symbol. Abbreviations: RANK, receptor activator of nuclear factor kappa-B; RANKL, receptor activator of nuclear factor kappa-B ligand; OPG, osteoprotegerin; TGF-β, transforming growth factor beta; OCL, osteoclasts; HSC, hematopoietic stem cells; pOCL, preosteoclasts; MCSF, macrophage colony-stimulating factor; Tregs, regulatory T cells.

**TABLE 7:** Estrogen representation in mathematical models of bone remodeling.

References	PMO treatment	Estrogen effects in the models
Explicit estrogen effects		
Rattanakul et al. (2003) ^3,‡^	Estrogen	Estrogen amplitude
		Increasing OCL removal rate
Schmidt et al. (2011) ^2^, Post et al. (2013) ^2^	Estrogen, tibolone, Ca placebo	Inhibiting OPG production rate
		Estrogen decay
		Estrogen production rates (endo and exogenous)
Berkhout et al. (2015) ^2^, Berkhout et al. (2016) ^2^	Ca placebo, bisphosphonates	Estrogen elimination rate
Chaiya and Rattanakul (2017) ^3^	Estrogen	Intermittent dosing
		First-order OCL degradation
		Zero-order OBL production
Javed et al. (2018) ^3,‡^	Denosumab	Inhibiting RANKL production
		Relative estrogen concentration
Jorg et al. (2022) ^3^	Bisphosphonates, RANKL antibodies, SCL antibodies, PTH analogs	Inhibiting OCL differentiation
		Inhibiting SCL secretion
		Stimulating OCL apoptosis
		Age-dependent estrogen concentration
Implicit estrogen effects		
Lemaire et al. (2004) ^2^	Parameter variations	Decreasing OPG production rate
Scheiner et al. (2013) ^2^, Larcher and Scheiner (2021) ^2^	-	Disease-modifying PTH production (dosage)
Scheiner et al. (2014) ^2^	Denosumab	Disease-modifying RANKL production
		Disease-modifying mechanical sensitivity
Trichilo et al. (2019) ^2^, Martin et al. (2019) ^2^	PTH	Disease-modifying RANKL production (dosage)
Lemaire and Cox (2019) ^2^	Denosumab, romosozumab	Decreasing OPG production rate
		Decreasing TGF-β production rate

The modeling approach is denoted by superscripts as follows: (1) power law, (2) mass action kinetics, or (3) neither. All models that follow the mass action kinetics approach include RANK, RANKL, OPG, and TGF-β. Models that do not follow this approach but include any of the signals above are indicated by the ^‡^ symbol. Abbreviations: RANK, receptor activator of nuclear factor kappa-B; RANKL, receptor activator of nuclear factor kappa-B ligand; OPG, osteoprotegerin; TGF-β, transforming growth factor beta; PMO, post-menopausal osteoporosis; OCL, osteoclasts; Ca, calcium; OBL, osteoblasts; SCL, sclerostin; PTH, parathyroid hormone.

In Section 2 we introduce the background of the biology for the bone local environment. Section 3 expands the background to include systemic biological and pharmacological influences on bone remodeling. Key techniques for mathematical modeling are categorized and introduced in Section 4 and are applied to cells of the bone remodeling cycle. Section 5 reviews existing biochemical models for bone remodeling. In Section 5.4 we emphasize how current models consider bone remodeling aspects from a systems biology perspective and point to several gaps in biological concepts that have yet to be considered thoroughly, thus highlighting opportunities for future systems biology models. Summaries of the mathematical models discussed in our review can be found in Supplementary Tables S1–S3, where the models are organized by modeling technique and include information about the cellular and biochemical molecules used, motivations and insights, and connections to other models.

## 2 Background on bone remodeling from a local perspective

Modern understanding of bone remodeling focuses locally on a basic multicellular unit (BMU) (Frost, 1966b; Frost, 1973; Jee, 2001; Allen and Burr, 2014). A BMU is considered a functional spatial packet where bone turnover occurs during remodeling (Frost, 1964a; Frost, 1966a). The prevalent view of the BMU typically consists of three cell types: osteoclasts, osteoblasts, and osteocytes. Osteoclast cells in the BMU are responsible for bone resorption, which involves the dissolution of the hydroxyapatite mineral layer and enzymatic degradation of the bone protein matrix (Bellido et al., 2014; Kenkre and Bassett, 2018). In opposition, osteoblast cells in the BMU form the bone protein matrix by depositing unmineralized tissue called osteoid, which undergoes a highly regulated mineralization process (Eriksen, 2010; Sims and Martin, 2020; Everts et al., 2022). Osteoblasts embedded in the osteoid tissue during this process differentiate into osteocyte cells. These osteocytes trigger and possibly terminate remodeling by releasing signaling molecules at various cycle phases (Bellido et al., 2014; Guder et al., 2020; Creecy et al., 2021).

### 2.1 Bone remodeling cycle

In its simplest form, a remodeling cycle consists of four phases: activation, resorption, formation, and resting. Bone remodeling is activated by localized mechanical damage and osteocyte apoptosis, which may be initiated by systemic biochemical changes, aging, or mechanical loading (Allen and Burr, 2014; Kenkre and Bassett, 2018; Pant et al., 2021). These factors trigger osteocytes to secrete signals that stimulate the proliferation of mononuclear cells, which fuse into preosteoclasts and then become active osteoclasts (Eriksen, 2010; Sims and Martin, 2020; Everts et al., 2022). As osteoclasts resorb bone, signaling factors (e.g., transforming growth factor beta (TGF-β), insulin-like growth factor (IGF)-1, IGF-2, bone morphogenic protein (BMP)2, and Wnt-10b (Sims and Martin, 2020)) are released from the bone matrix or secreted by osteoclasts themselves. These signals, in turn, initiate osteoblast proliferation, migration, and activation. Osteoblasts produce the extracellular protein matrix that becomes bone tissue. Embedded osteocytes secrete signals to slow bone formation and indicate when the resorption cavity is filled, leading to a resting phase.

**FIGURE 1:**
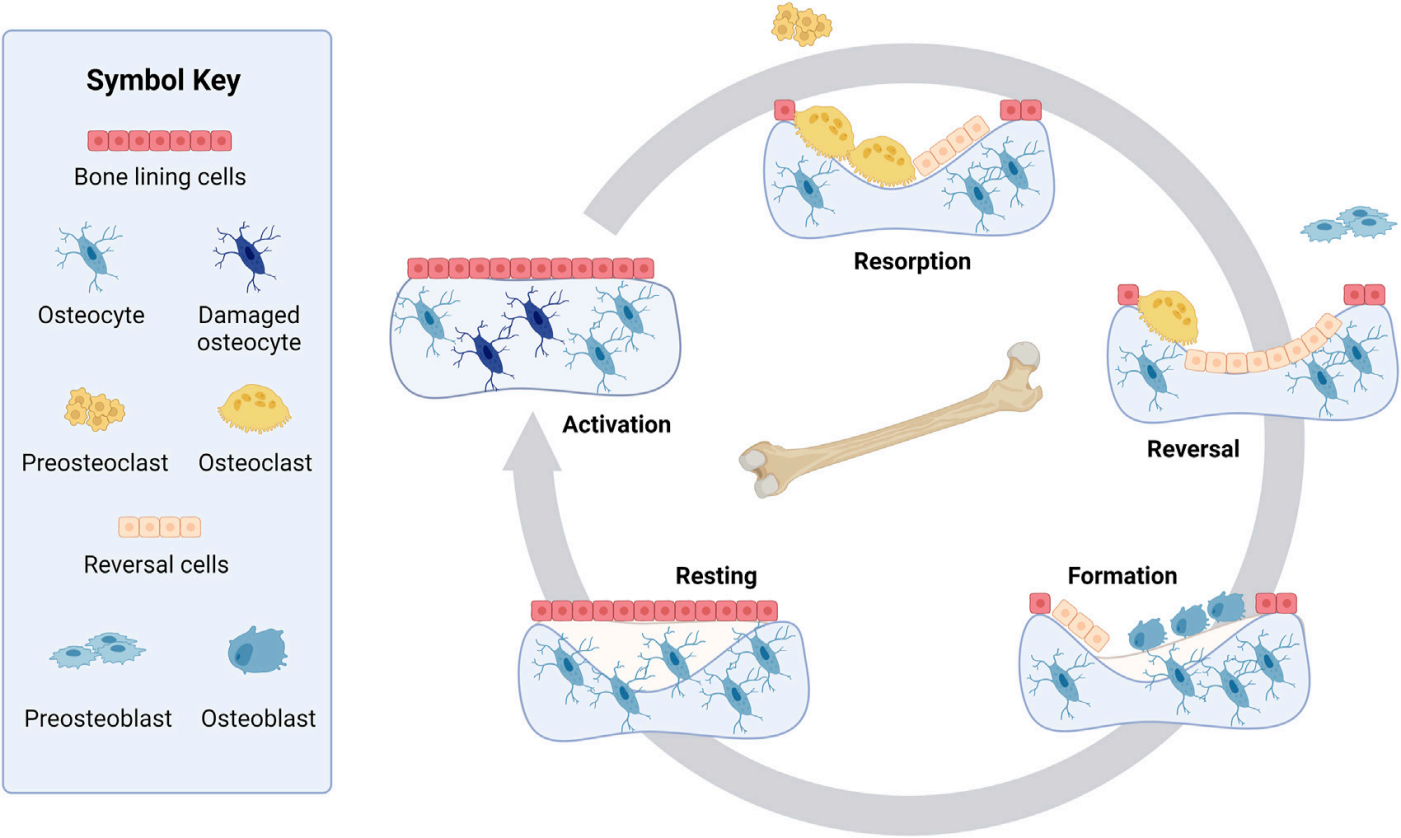
Bone remodeling cycle. Resting bone is covered in bone lining cells with healthy osteocytes embedded in the bone. Step 1, Activation: Bone remodeling starts when the osteocytes are activated or damaged. Step 2, Resorption: During the resorption phase, osteoclasts are formed from preosteoclasts and break down bone in a cavity. Step 3, Reversal: Mononuclear cells that are known as reversal cells prepare the surface as preosteoblasts arrive at the cavity during the reversal phase. These preosteoblasts proliferate and convert into osteoblasts. Step 4, Formation: Osteoblasts reform the bone matrix by depositing osteoid, which later mineralizes. While the matrix is being deposited, some osteoblasts embed in the bone, becoming osteocytes. Step 5, Resting: The bone remains resting until another cycle of bone remodeling is initiated. Created with BioRender.com.

A more complex and recent representation of the bone remodeling cycle adds a reversal phase between the resorption and formation phases (Jee, 2001; Allen and Burr, 2014; Bellido et al., 2014) (Figure 1). Before osteoblasts rebuild bone, it is suggested that the resorbed bone cavity is cleared of debris by reversal cells, which are currently not considered part of the BMU (Delaisse et al., 2020). The origin of these cells is unclear, but they express markers of osteoblastic lineage (Delaisse et al., 2020; Epsley et al., 2021). Bone lining cells are another cell not canonically considered part of the BMU. However, osteoblasts can also become bone lining cells at the end of the bone formation phase, forming a protective layer on the bone surface that prevents osteoclasts from interacting with bone where remodeling should not occur (Bellido et al., 2014; Florencio-Silva et al., 2015; Della Corte et al., 2020).

### 2.2 Cells of the BMU

**FIGURE 2:**
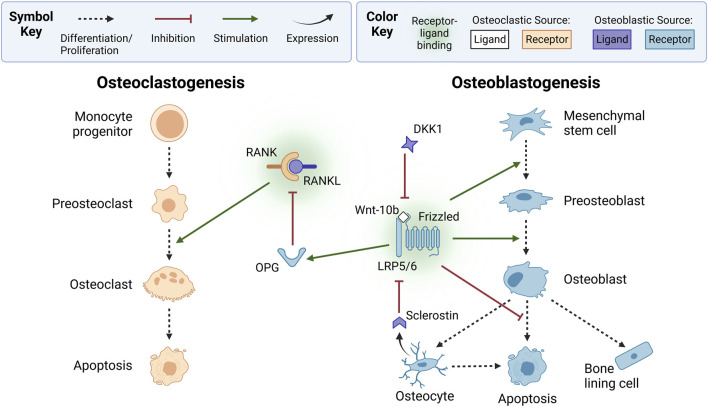
Osteoclasts and osteoblasts form via osteoclastogenesis and osteoblastogenesis, respectively. In osteoclastogenesis, osteoclasts are derived from monocyte progenitor cells that differentiate into mononuclear preosteoclasts, which fuse into active multinucleated osteoclasts. Preosteoclast proliferation and fusion is stimulated by osteoblastic lineage-derived receptor activator of nuclear factor kappa B ligand (RANKL) binding to RANK on osteoclastic cells. Osteoblast-produced osteoprotegerin (OPG), a decoy receptor, inhibits osteoclastogenesis by binding to RANKL. In osteoblastogenesis, osteoblasts originate from mesenchymal stem cells that differentiate into preosteoblasts. Osteoblastogenesis is typically stimulated by canonical wingless-related integration site (Wnt) signaling, which occurs when osteoclast-derived Wnt-10b ligands bind to lipoprotein receptor-related protein 5 or 6 (LRP5/6) and Frizzled coreceptors on osteoblastic cells. Canonical Wnt signaling also stimulates OPG expression and inhibits osteoblast apoptosis. Osteoblastogenesis is inhibited by osteoblast-derived dickkopf-related protein 1 (DKK1) and osteocyte-derived sclerostin, which bind to canonical Wnt LRP5/6 receptors. Receptors and ligands expressed from osteoclastic or osteoblastic sources are not explicitly shown with arrows to simplify this diagram; instead, they are indicated by color. Ligands from osteoclastic sources include Wnt-10b (white). Receptors from osteoclastic sources include RANK (orange). Ligands from osteoblastic sources include RANKL, sclerostin, and DKK1 (purple). Receptors from osteoblastic sources include OPG, LRP5/6, and Frizzled (blue). Created with BioRender.com.

Osteoclasts are the only cells known to break down bone. They originate from hematopoietic stem cells that differentiate into monocyte progenitors (Bellido et al., 2014) (Figure 2). In bone remodeling, the monocyte progenitor cells are often called uncommitted osteoclasts because they can also differentiate into other cell types. Upon stimulation by various signaling factors, monocyte progenitor cells become mononuclear preosteoclasts (also known as precursor osteoclasts) that later proliferate and fuse into osteoclasts (Martin and Rodan, 2009; Eriksen, 2010; Bellido et al., 2014; Kim et al., 2020; Sims and Martin, 2020; Epsley et al., 2021; Everts et al., 2022). Thus, osteoclasts are multinucleated cells that remove bone (Jee, 2001).

Osteoblasts produce osteoid, the collagenous organic matrix that makes up bone (Allen and Burr, 2014; Bellido et al., 2014; Sharma et al., 2020). Osteoblasts are derived from mesenchymal stem cells from bone marrow that differentiate into osteochondro progenitor cells (Figure 2). These are often classified as uncommitted osteoblasts. Osteochondro progenitor cells later differentiate into committed preosteoblast cells (also known as precursor osteoblasts). During bone remodeling, signaling factors activate the proliferation and migration of preosteoblasts to the resorption site, where they differentiate into osteoblast cells. When osteoblasts become trapped in the osteoid collagen matrix, they differentiate into osteocytes. The osteoblasts that remain after bone formation become inactive bone lining cells or undergo apoptosis (Jee, 2001; Bellido et al., 2014).

Although questions remain about what regulates osteoblast differentiation into osteocytes, the change is marked by the formation of dendrites (Creecy et al., 2021) that form a network to communicate with other osteocytes and bone cells (Bellido et al., 2014). Osteocytes have a 25-year lifespan and are the longest-living and most abundant bone cells (Jee, 2001; Bonewald, 2011; Bellido et al., 2014; Florencio-Silva et al., 2015).

The roles of osteocytes in the bone remodeling process are relatively recent discoveries, as these cells were initially considered inert (Bonewald and Johnson, 2008; Bonewald, 2011; Florencio-Silva et al., 2015). Osteocytes stimulate remodeling in response to mechanical and hormonal stimuli and other stressors (Bonewald, 2011; Bellido et al., 2014) by secreting key regulatory molecules for cellular differentiation and activity in the BMU (Ait Oumghar et al., 2020; Creecy et al., 2021) and regulate calcium homeostasis by triggering mineral release from the bone matrix (Bonewald, 2011; Jähn et al., 2017). Osteocyte apoptosis following estrogen deficiency increases remodeling (Tomkinson et al., 1997; Tomkinson et al., 1998; Emerton et al., 2010; Khosla et al., 2012; Bellido et al., 2014). Osteocyte lifespan and apoptosis are also linked to *in vivo* mechanical forces and accumulated microdamage in aging (Bellido et al., 2014).

As with most biological concepts, the bone remodeling process is more complex than a four-step process consisting of only three cell types. The reversal phase is an example of such complexity. Precursor bone cells are another example. Although not included in the simplified BMU, precursor bone cells are important cells in the bone remodeling cycle (Boyle et al., 2003; Rutkovskiy et al., 2016). The numerous signaling factors that regulate bone remodeling add another layer of complexity (Figure 2), discussed further in Sections 2.3, 3.

### 2.3 Signaling pathways of the BMU

A key signaling mechanism driving the coordination of osteocytes, osteoclasts, and osteoblasts is the RANK-RANKL-OPG pathway (Figure 2). Preosteoclasts and active osteoclasts express receptor activator of nuclear factor kappa-B (RANK) (Eriksen, 2010). RANK binds to its ligand RANKL, a soluble and membrane-bound protein expressed by osteoblastic-lineage cells, such as mesenchymal stem cells, preosteoblasts, osteoblasts, and osteocytes (Eriksen, 2010). RANK-RANKL binding triggers intracellular cascades, such as the nuclear factor kappa B (NF-*κ*B) pathway, which produces nuclear factor of activated T cell cytoplasmic 1 (NFATc1), a transcription factor that induces osteoclastic genes (Walsh and Choi, 2014). These genes regulate cell proliferation, differentiation, and survival through the osteoclastic lineage, a process called osteoclastogenesis. RANK-RANKL binding is inhibited by osteoprotegerin (OPG), a soluble decoy receptor expressed by osteoblastic cells that binds to RANKL (Eriksen, 2010).

As shown in Figure 2, the wingless-related integration site (Wnt) pathways play a complementary role in bone remodeling by regulating osteoblastogenesis (Bennett et al., 2005; Bennett et al., 2007). Wnt is a family of 19 glycoproteins that can activate the canonical Wnt/β-catenin pathway, the non-canonical Wnt/Ca^2+^ pathway, and the Wnt/planar cell polarity pathway (Bonewald and Johnson, 2008; Houschyar et al., 2019; Maeda et al., 2019). Wnt ligands, such as osteoclast-derived Wnt-3a and Wnt-10b, activate the canonical pathway by binding to low-density lipoprotein receptor-related protein 5 or 6 (LRP5/6) and the Frizzled coreceptor (Lerner and Ohlsson, 2015; Maeda et al., 2019; Perkins et al., 2023). This increases β-catenin levels, upregulating osteoblastic genes (Perkins et al., 2023). The canonical pathway promotes mesenchymal stem cell differentiation into preosteoblasts by inhibiting their differentiation into adipocytes and chondrocytes (Siddiqui and Partridge, 2016; Maeda et al., 2019; Kim et al., 2020). Additionally, canonical signaling upregulates OPG expression by osteoblastic-lineage cells (Kramer et al., 2010; Bellido et al., 2014; Plotkin and Bivi, 2014), suppressing osteoclastogenesis (Siddiqui and Partridge, 2016). The canonical cascade is inhibited by osteoblast-derived dickkopf-related protein 1 (DKK1) and osteocyte-derived sclerostin, which bind to LRP5/6 instead of Wnt ligands (Maeda et al., 2019). Osteocytes secrete sclerostin to terminate and prevent activation of a remodeling cycle (Eudy et al., 2015; Ait Oumghar et al., 2020; Creecy et al., 2021).

As in the Wnt/β-catenin pathway, non-canonical signaling stimulates osteoblastogenesis when osteoclast-derived Wnt binds to osteoblastic receptors (Lerner and Ohlsson, 2015). Contrarily, non-canonical signaling can inhibit or stimulate osteoclastogenesis (Lerner and Ohlsson, 2015). Osteoblast-derived Wnt-16 inhibits osteoclast differentiation directly by activating osteoclastic receptors. However, it indirectly stimulates osteoclastogenesis by activating osteoblastic receptors that upregulate OPG production (Kim et al., 2020). Together, these findings highlight the complexity of Wnt signaling and its regulation of bone remodeling. In the remainder of this paper, we use Wnt and Wnt signaling to refer to the canonical Wnt/β-catenin pathway, unless otherwise specified. For further details on chemical agents that regulate cells of the BMU organized by cell type, readers are referred to Jee (2001).

## 3 Background on bone remodeling from a systemic perspective

**FIGURE 3:**
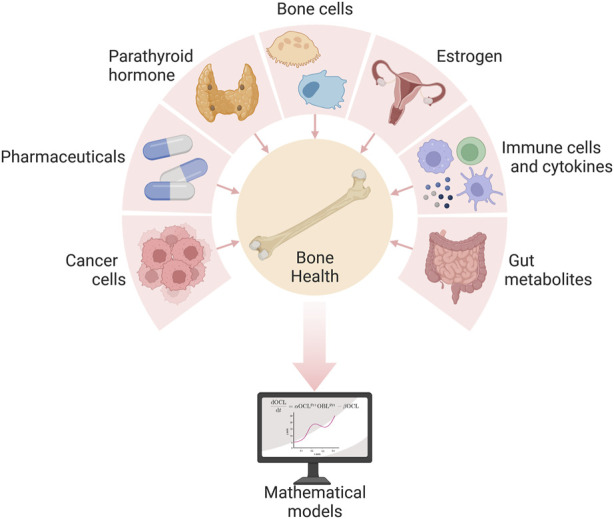
Several local and systemic cells and chemicals influence bone health, and their complex interactions can be explored via mathematical models of the bone remodeling process. Created with BioRender.com. Adapted from “Pie Chart 7X” by BioRender.com (2022). Retrieved from https://app.biorender.com/biorender-templates.

The numerous signals that regulate bone remodeling originate not only from bone cells (Figure 2) but also from beyond the bone microenvironment (Figure 3). Systemic influences on bone remodeling are seen in multiple bone diseases. Rheumatoid arthritis, for example, is an autoimmune condition that causes joint inflammation and destruction but also increases the risk of osteoporosis twofold (Haugeberg et al., 2000; Hauser et al., 2014; Llorente et al., 2020). This hints at immune-bone crosstalk. Furthermore, sex hormones have long been thought to control the bone remodeling process due to the link between estrogen decline and postmenopausal osteoporosis (Rattanakul et al., 2003; Li et al., 2016; Ait Oumghar et al., 2020; Lehmann et al., 2021). Sex hormones also regulate the immune system (Kovats, 2015). Bone cancers (e.g., osteosarcoma) and metastatic bone disease also interfere with bone homeostasis (Buenzli et al., 2012b; Araujo et al., 2014; Ji et al., 2014; Farhat et al., 2017). Intestinal dysbiosis also influences the bone remodeling cycle (Li et al., 2016; Zaiss et al., 2019; Hao et al., 2021). The complexity of bone remodeling extends beyond the local bone environment to the systemic whole body level (Figure 3). The rest of this section provides an overview of several local and systemic cellular and chemical signaling mechanisms that modulate bone remodeling.

### 3.1 Osteoimmunology

Evidence that immune activity modulates bone remodeling first appeared in Horton et al. (1972) (Takayanagi, 2007; Della Corte et al., 2020). This study showed that bone cultures from rats had increased resorption activity after treatment with supernatant from cultures with human peripheral blood mononuclear cells. This was an early sign of crosstalk between bone and immune cells. However, the importance of bone-immune interplay was not fully realized until multiple publications in the 1990s showed that signals from the immune system signal bone remodeling (Iotsova et al., 1997; Wong et al., 1997; Fuller et al., 1998; Dougall et al., 1999; Kotake et al., 1999; Naito et al., 1999; Suda et al., 1999). One such study found that RANK, a protein of the tumor necrosis factor (TNF) superfamily secreted by immune cells, is a crucial receptor in bone remodeling (Dougall et al., 1999). Mice lacking this receptor protein had fewer B cells in the spleen, almost no peripheral lymph nodes, and fewer mature osteoclasts. The importance of these discoveries is highlighted by Arron and Choi (2000). This seminal article coined the term osteoimmunology to describe the intersection of bone and immune research, leading to a new subfield of research. Several more recent reviews provide in-depth surveys of osteoimmunology beyond the scope of the present review (Lerner, 2006; Eastell et al., 2016; Weitzmann and Ofotokun, 2016; Weitzmann, 2017; Dar et al., 2018; Ponzetti and Rucci, 2019). Several cytokines and immune cells influence bone remodeling and are summarized in the following.

#### 3.1.1 MCSF, TGF-β, and other cytokines

In addition to Wnt and RANK-RANKL-OPG signaling, two cytokines play essential signaling roles in bone remodeling: macrophage colony-stimulating factor (MCSF) and TGF-β. MCSF stimulates osteoclastogenesis by binding to monocyte progenitor cells and preosteoclasts. This triggers intracellular cascades that induce NFATc1, the main transcription factor for osteoclastogenesis (Guder et al., 2020). The importance of MCSF stems from its role in stimulating the first stage of osteoclastogenesis (Figure 2), which RANKL does not stimulate, and the proliferation of osteoclast precursor cells. The role of TGF-β is less straightforward. Its regulatory effects are concentration dependent (Janssens et al., 2005; Wu et al., 2016). Moreover, it regulates both bone formation and bone resorption. Low concentrations of TGF-β stimulate osteoclast production while promoting preosteoblast migration and proliferation. High concentrations inhibit osteoclastogenesis. Also, high concentrations inhibit preosteoblast migration and late-stage osteoblast differentiation. Although these contradictory findings still puzzle researchers, the mechanism of changes in TGF-β concentration during remodeling is well understood. Inactive TGF-β is stored in the extracellular matrix of bone (Epsley et al., 2021). As osteoclasts remove bone, TGF-β is released and activated, increasing the concentration of TGF-β (Janssens et al., 2005; Matsumoto and Abe, 2011).

Numerous other cytokines also regulate bone remodeling. TGF-β is not the only cytokine released from the bone matrix during resorption; others include IGF-1, IGF-2, and BMP2 (Sims and Martin, 2020). Typically, cytokines are classified as osteoclastogenic or osteoblastogenic, though their roles may be concentration-dependent as described with TGF-β. Bone resorption is inhibited by anti-inflammatory cytokines such as interleukin (IL)-4, IL-10, IL-13, IL-18, and interferon (IFN)-γ (Walsh et al., 2006). Conversely, it is stimulated by pro-inflammatory cytokines such as IL-1, IL-1β, TNF-α, IL-6, IL-11, IL-15, and IL-17. These cytokines modulate RANKL and Wnt signaling to increase osteoclast activity (Walsh et al., 2006; Tilg et al., 2008). For example, TNF-α upregulates the expression of RANKL, DKK1, and sclerostin in osteocytes (Kitaura et al., 2020; Epsley et al., 2021). The influx of RANKL promotes osteoclastogenesis, while the influx of DKK1 and sclerostin inhibits osteoblastogenesis. Cytokines interact in a complex network with RANK-RANKL-OPG, Wnt, MCSF, and TGF-β to regulate bone remodeling.

For further detailed coverage of cytokines and growth factors that locally regulate bone cell function and thorough diagrams of signaling ligands and their associated receptors, intracellular kinases and transcription factors, and biological outcomes, readers are referred to Plotkin and Bivi (2014).

#### 3.1.2 Immune cells

Immune cells contribute to bone homeostasis through cytokine expression and direct immune cell activity. Osteoclasts are derived from innate immune cells called monocytes (Saxena et al., 2021). Monocytes are more commonly differentiated into macrophages and dendritic cells. Studies have shown that these cells can transdifferentiate into preosteoclasts (Bonomo et al., 2016; Saxena et al., 2021; Srivastava and Sapra, 2022). Macrophages further modulate bone remodeling through the expression of inflammatory cytokines IL-1, IL-6, and TNF-α or bone formation factors IL-10, BMP-2, and TGF-β (Fischer and Haffner-Luntzer, 2022). In contrast, dendritic cells primarily stimulate osteoclastogenesis through RANK-RANKL activation of T cells, which upregulates T cell production of RANKL, IL-1, IL-6, IL-17, and TNF-α (Bonomo et al., 2016). However, not all T cells are osteoclastogenic.

Different populations of T cells affect osteoclasts and osteoblasts in different ways. Naïve CD4^+^ T cells can differentiate into osteoclastogenic subtypes, e.g., T helper (Th)17 and Th9 cells, or anti-osteoclastic subtypes, e.g., Th1, Th2, and T regulatory (Treg) cells, characterized by their cytokine expression profiles (Guder et al., 2020). For example, Th17 cells express high levels of IL-17, which upregulates RANK in preosteoclasts and RANKL in osteoblasts, increasing bone resorption (Fischer and Haffner-Luntzer, 2022; Srivastava and Sapra, 2022). Th17 cells also secrete IL-6, RANKL, and TNF-α to promote osteoclastogenesis and suppress osteoblast activity (Srivastava et al., 2018; Epsley et al., 2021). Cytokine profiles of Th1, Th2, and Treg cells contrast the profiles from Th17 cells. These cells secrete anti-osteoclastic cytokines such as IFN-γ, IL-4, TGF-β1, and IL-10 (Okamoto et al., 2017; Srivastava et al., 2018; Guder et al., 2020). However, following the pattern of Wnt, TGF-β, and other cytokines, T cell roles are not always clear. Activated Tregs secrete DKK1, which inhibits Wnt-mediated bone formation (Lehmann et al., 2021). This inhibitory effect contrasts studies showing Tregs increase Wnt-10b production by CD8^+^ T cells (Tyagi et al., 2018). Despite this, studies indicate a balance between Th17 and Treg cells is important for healthy bone remodeling, such that higher Th17 to Treg ratios contribute to rheumatoid arthritis and osteoporosis (Okamoto et al., 2017; Srivastava et al., 2018). Declining bone health is associated with many classic inflammatory diseases, such as periodontitis, rheumatoid arthritis, and aseptic prosthesis (Epsley et al., 2021). To obtain a more complete picture of bone remodeling, it is vital to consider these complex bone-immune interactions.

### 3.2 Endocrine system and pharmaceuticals

**FIGURE 4:**
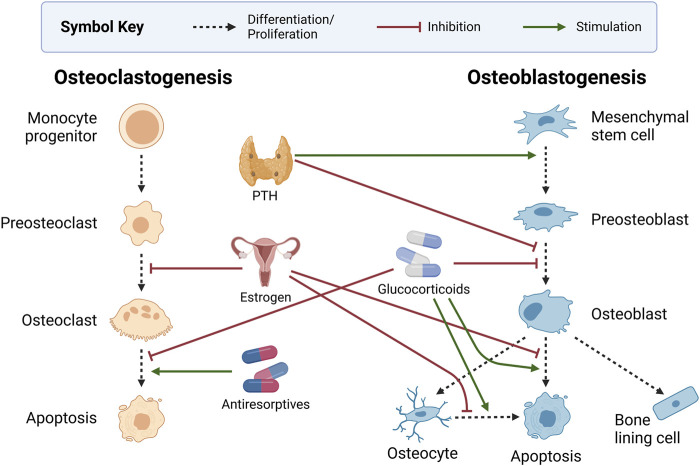
Endocrine and pharmaceutical modulators of bone health. Intermittent dosing of parathyroid hormone (PTH) stimulates preosteoblast formation and inhibits preosteoblasts’ differentiation to osteoblasts. Estrogen inhibits the development of osteoclasts while also protecting osteoblasts and osteocytes from apoptosis. Glucocorticoids inhibit osteoblast development and survival, increase osteocyte apoptosis, and decrease osteoclast apoptosis. Antiresorptives such as bisphosphonates and monoclonal antibodies promote osteoclast apoptosis. Created with BioRender.com.

Figure 4 highlights the influence of the endocrine system and other common bone-related medications on bone health. The cross-talk between the endocrine and the skeletal systems is expansive. Here, we discuss only parathyroid hormone (PTH) and estrogen, which are most prevalent in bone mathematical research. Intermittent PTH and hormone replacements for estrogen are commonly used as pharmacological interventions for bone diseases. As such, we consider pharmaceuticals together with the endocrine system.

#### 3.2.1 Parathyroid hormone

PTH is a systemic hormone that regulates calcium levels in the blood in part by triggering calcium release from the bone. Chief cells within the parathyroid gland produce PTH when serum calcium levels are low (Chaiya and Rattanakul, 2017). The increase in circulating PTH triggers bone remodeling, and subsequently, osteoclasts release calcium from the bone into the blood to maintain homeostasis (Peterson and Riggs, 2010; Coelho et al., 2016). This ability to stimulate remodeling has led to the development of synthetic PTH for osteoporosis treatment. However, PTH is another signaling factor with a dual role in osteoclastogenesis. Circulating PTH stimulates osteoclast activity by increasing the RANKL to OPG ratio but inhibits osteoclast formation by decreasing sclerostin and DKK1 (Silva and Bilezikian, 2015; Kenkre and Bassett, 2018; Wein and Kronenberg, 2018; Guder et al., 2020). Exogenous PTH alters bone remodeling differently depending on the administration schedule. Continuous administration decreases overall levels of bone density, whereas intermittent administration increases bone density levels (Coelho et al., 2016; Lemaire and Cox, 2019). This has led many researchers to develop mathematical models to understand the mechanisms of PTH regulation.

The dual role of PTH is currently understood to involve both stimulation of preosteoblast production and inhibition of preosteoblast differentiation (Chaiya and Rattanakul, 2017). As a result, preosteoblast cell populations increase while osteoblast populations remain unchanged. This causes an increase in the RANKL to OPG ratio since osteoblastic cells produce more OPG and less RANKL (Gori et al., 2000) as they mature. A short burst of PTH stimulates remodeling by increasing RANKL and suppressing OPG. High concentrations of PTH over a long period, as in the case of hyperparathyroidism, dysregulate bone remodeling due to the overproduction of osteoclasts. This leads to a larger resorption cavity that the limited number of osteoblasts cannot fill. This interrelationship between bone cells and PTH exemplifies the complexity of the bone remodeling process.

#### 3.2.2 Estrogen

Estrogen and bone health have been closely linked for decades due to the correlation between postmenopausal estrogen decline and bone loss (Khosla et al., 2012). Although early research on the mechanism of estrogen regulation of bone remodeling was unclear, recent studies in osteoimmunology have improved our understanding. Estrogen deficiency increases bone turnover and unbalanced remodeling (Khosla et al., 2012). This occurs through estrogen-mediated inhibition of RANKL production and stimulation of OPG expression, which limits osteoclastogenesis (Eriksen, 2010; Florencio-Silva et al., 2015; Noirrit-Esclassan et al., 2021). Estrogen has also been shown to prevent apoptosis of osteoblasts and osteocytes (Florencio-Silva et al., 2015). This is consistent with studies showing that estrogen deficiency induces osteocyte apoptosis (Tomkinson et al., 1997; Tomkinson et al., 1998; Emerton et al., 2010; Khosla et al., 2012; Delgado-Calle and Bellido, 2022). Ovariectomized (OVX) murine experiments demonstrate that estrogen directly supports bone formation by upregulating Wnt-10b in bone marrow stromal cells (Perkins et al., 2023).

Further evidence of the estrogen-bone link is based on the presence of estrogen receptors (ER) on bone cells and targeted deletion studies. ERα is found on osteoclastic and osteoblastic cells, while ERβ is found on osteoblasts (Sharma et al., 2020). Targeted deletion of osteoblastic ERα in murine models led to low bone mass in both males and females (Almeida et al., 2017; Gao et al., 2021). The targeted deletion of ERα in osteoclasts and osteoclast progenitor cells increased osteoclast numbers in females but not in males (Almeida et al., 2017). Another study of ERβ deletion in mesenchymal stem cells found that bone mass increased only in female rodents (Almeida et al., 2017). These knockout studies indicate that estrogen signaling is vital to bone homeostasis in males and females, with sex-based differences in these signaling mechanisms.

Estrogen regulates bone remodeling through direct and immune-mediated mechanisms (Khosla et al., 2012). For instance, estrogen protects against T-cell-mediated bone loss by upregulating Wnt signaling. While mice with DKK1-expressing T cells experienced OVX-induced bone loss, knockout mice without DKK1-expressing T cells did not, and prior to OVX, these mice exhibited higher bone mass (Lehmann et al., 2021). The loss of bone in response to estrogen deficiency is recognized as a cytokine-driven process involving T cell populations such as Tregs and Th17 cells that results in the bone resorption activity of osteoclasts exceeding that of bone-forming osteoblasts (Pacifici, 2012). The anti-inflammatory effect of estrogen extends to macrophages and dendritic cells. Estrogen deficiency has been shown to induce the transdifferentiation of pro-inflammatory M1 macrophages into osteoclasts and increase the ratio of M1 to anti-inflammatory M2 macrophages (Saxena et al., 2021). Without estrogen, more dendritic cells were shown to express IL-7 and IL-15, which upregulates IL-17 and TNF-α production by T cells (Saxena et al., 2021). Furthermore, a cross-sectional clinical study of postmenopausal women showed that elevated inflammatory markers such as IL-6, IL-β, and TNF-α were negatively correlated with bone mass (Damani et al., 2023). These findings indicate estrogen regulates bone remodeling through immune-mediated effects and direct signaling within the bone microenvironment.

#### 3.2.3 Pharmaceuticals

Pharmaceuticals can indirectly regulate remodeling while treating various diseases, or they can be designed to target mechanisms in the bone remodeling cycle intentionally. Glucocorticoids are anti-inflammatory agents that are used broadly but have negative effects on bone health by decreasing osteoblast and osteocyte populations and increasing osteoclast survival (Hardy et al., 2018). Despite this, they are often used to reduce chronic inflammation in rheumatoid arthritis that can otherwise lead to osteoporosis. Teriparatide, a PTH analog that has anabolic effects on the bone, has several proposed mechanisms for its action (Wein and Kronenberg, 2018). Since teriparatide requires expensive daily injections, it is used mainly for severe osteoporosis or those who need to use glucocorticoids long-term for other conditions (Hodsman et al., 2005).

Estrogen replacement therapy (ERT) is a pharmaceutical intervention directly designed to impact bone remodeling based on the association between estrogen decline and osteoporosis after menopause. Hormone replacement therapy (HRT) augments estrogen with progestogens. Selective estrogen receptor modulators (SERMS) act as estrogen receptor agonists in some tissues like bone and antagonists in other tissues, sometimes detrimentally (Ellis et al., 2015). These treatments have different and controversial risks associated with breast cancer, coronary heart disease, and stroke that impact their adoption based on individualized management of benefits and risks (Manson et al., 2013; Ellis et al., 2015; Hodis and Sarrel, 2018; Faubion et al., 2022; Onwude, 2022; Nudy et al., 2023).

Another group of medications to reduce bone loss are antiresorptives, which target signaling mechanisms of bone remodeling that contribute to osteoclast activity. Bisphosphonates are antiresorptives that are currently the most common treatments for bone loss. These drugs inhibit bone resorption by inducing osteoclast apoptosis and reducing osteoclast activity (Berkhout et al., 2015; Coelho et al., 2016; Aibar-Almazán et al., 2022). Bisphosphonates even alter bone remodeling after treatment is terminated because they bind to hydroxyapatite crystals on the surface of the bone matrix (Drake et al., 2008; Aibar-Almazán et al., 2022). They can be released from the surface in subsequent remodeling cycles (Coelho et al., 2016). Bisphosphonates are generally well tolerated but are most often discontinued due to gastrointestinal distress or concerns about side effects such as osteonecrosis of the jaw or spiral fractures of the femur midshaft (Aibar-Almazán et al., 2022). The monoclonal antibody denosumab is a newer antiresorptive. Denosumab inhibits resorption by blocking RANK-RANKL binding. It acts as an OPG mimic, binding to RANKL to prevent osteoclast activation. Although denosumab is more effective at preventing bone loss than bisphosphonates and used for metastatic cancers that target bone, there is a higher risk of osteonecrosis of the jaw (Aibar-Almazán et al., 2022; Fu et al., 2023) and higher frequency of second tumors in cancer patients on denosumab (Stopeck et al., 2010; Fizazi et al., 2011; Henry et al., 2011; Raje et al., 2018; Tovazzi et al., 2019).

Romosozumab is the newest pharmaceutical intervention for the bone remodeling cycle. Romosozumab is a monoclonal antibody that binds to sclerostin, allowing Wnt ligands to activate the canonical pathway, stimulate bone formation, and inhibit bone resorption (Figure 2). The disadvantage is that romosozumab is associated with more undesirable side effects than bisphosphonates including increased risk of adverse cardiovascular events (Asadipooya and Weinstock, 2019; Aibar-Almazán et al., 2022). Overall, targeted treatments of the bone remodeling cycle have poor compliance and high discontinuation rates due to a combination of high costs, unwanted side effects, and psychological factors (Aibar-Almazán et al., 2022). Viable new treatments need to eliminate or reduce these concerns.

### 3.3 Gut metabolites and immune connections

Gut and bone health are connected via shared crosstalk with the immune system. The gut regulates immune response and bone remodeling through the intestinal barrier. The intestinal barrier consists of a mucus layer and tight junction proteins, which protect the immune system from pathogens and toxins (Paone and Cani, 2020). Intestinal microbes help maintain this barrier (Anderson et al., 2010; Sjögren et al., 2012). Sjögren et al. (2012) found that conventional mice had increased gut permeability and inflammatory cytokines, resulting in lower bone mass than germ-free mice. Since systemic immune inflammation can increase bone resorption, it follows that gut-induced immune inflammation can cause bone loss. Additionally, estrogen deficiency compromises the gut barrier, affecting inflammation onset and trafficking of immune cells from the gut to the periphery (Braniste et al., 2009; Li et al., 2016; Rios-Arce et al., 2017).

Gut microbial populations contribute to gut-mediated immunomodulation of bone health through metabolites such as short-chain fatty acids (SCFAs). SCFAs stimulate mucus production and tight junction protein expression (Gizard et al., 2020; Paone and Cani, 2020; Arnold et al., 2021). Additionally, SCFAs can enter the bloodstream, where they not only inhibit NF-κB pathways and downregulate TNF-α but also upregulate macrophage and dendritic cell expression of IL-10 (Hosseinkhani et al., 2021). In a study of healthy male mice, dietary supplementation with the SCFA butyrate showed increases in *Clostridia* populations, circulating Tregs, Wnt-10b, osteoblastogenesis, and bone mass (Tyagi et al., 2018). Chen et al. (2020) also showed increased SCFAs and Tregs due to prebiotic lactulose administration in OVX mice, preventing subsequent bone loss. Furthermore, SCFAs have been shown to improve calcium absorption, balance Tregs and Th17 cells, and produce bone-forming IGF-1 (Behera et al., 2020; Lu et al., 2021; Perkins et al., 2023). Another study demonstrated that a change in microbial composition reduced SCFAs, increased gut permeability, increased serum lipopolysaccharide (an inflammatory marker), and, ultimately, increased osteoclast activity, leading to bone loss (Behera et al., 2020). Further studies provided evidence in support of probiotic and prebiotic restoration of intestinal barrier function and prevention of bone loss (Schepper et al., 2019; Chen et al., 2020). The numerous osteogenic functions of SCFAs thus indicate their potential for treating inflammatory bone loss.

Evidence linking gut health and inflammation has led researchers to explore opportunities for dietary prebiotic and probiotic treatment of estrogen-deficient bone loss (Sjögren et al., 2012; Britton et al., 2014; Li et al., 2016). Dietary manipulation of the gut microbiota using probiotics (e.g., *Lactobacillus* and *Bifidobacteria*) protected against bone loss in a small clinical trial (Takimoto et al., 2018) and in animal models of periodontal disease (Messora et al., 2013), diabetes (Zhang et al., 2015), and estrogen deficiency (Britton et al., 2014; Ohlsson et al., 2014; Li et al., 2016). Li et al. (2016) induced sex hormone deficiency in germ-free and conventional mice. They found that conventional mice had degraded intestinal walls, increased immune inflammation, and increased bone loss compared to germ-free mice. Their treatment of conventional mice with probiotics prevented inflammation and bone loss. Mechanistically, another study demonstrated that probiotic treatment of mice with drug-induced osteoporosis increased Wnt-10b levels (Perkins et al., 2023). Consumption of SCFAs and prebiotics, which can be fermented to form SCFAs, also increased intestinal calcium absorption in adolescents and post-menopausal osteoporosis patients (Behera et al., 2020; Lu et al., 2021). Other murine studies indicated that prebiotic and probiotic treatments prevented OVX-induced increases in Th17 cells and the inflammatory cytokines IL-17, TNF-α, IL-6, and RANKL (Lu et al., 2021). These changes were accompanied by reduced intestinal permeability and increases in IL-10, IGF-1, and BMPs that promote osteoblastogenesis and improve bone strength (Behera et al., 2020). Additional studies linked prebiotics, such as oligosaccharides, to altered SCFAs, enhanced intestinal barrier function, and programmed tolerogenic immune cell responses (Chonan et al., 1995; Abrams et al., 2005; Weaver et al., 2011; Legette et al., 2012; Arpaia et al., 2013; Furusawa et al., 2013; Smith et al., 2013; Chang et al., 2014; Singh et al., 2014; Tan et al., 2016; Chen et al., 2017; Hu et al., 2018; Ghosh et al., 2021). Numerous studies including several of our own showed how foods with prebiotic activity affect SCFAs, the immune system, and the bone even without alterations in gut barrier function or where there is no compromise in gut barrier function (Chonan et al., 1995; Roberfroid et al., 2002; Arjmandi et al., 2004; Abrams et al., 2005; Scholz-Ahrens et al., 2007; Bu et al., 2009; Weaver et al., 2011; Legette et al., 2012; Vulevic et al., 2008, 2015; Rendina et al., 2012; Smith et al., 2014; Ojo et al., 2016, 2019, 2021; Graef et al., 2018a,b; Shen et al., 2019; Smith et al., 2019; Dodier et al., 2021; Keirns et al., 2020; Smith et al., 2022).

### 3.4 Metastatic cancer cells

Many cancers metastasize to bone, including prostate, breast, and myeloma cancers (Ait Oumghar et al., 2020; Coleman et al., 2020). Cancer cells dysregulate the bone remodeling cycle by secreting osteoclastogenic cytokines that initiate bone resorption to make room for tumor growth (Marathe et al., 2008). This increased remodeling leads to increased bone formation during the early stages of tumor growth (Ayati et al., 2010). However, continued remodeling results in a tumor-initiated resorption rate that exceeds that of bone formation. It also increases the rate of tumor growth, which is stimulated by the TGF-β released from the bone matrix during resorption. Eventually, the growing tumor fills the resorption cavity before osteoblast signaling occurs (Ji et al., 2014). Cancer cells also secrete molecules besides cytokines to promote bone resorption or inhibit bone formation. For example, myeloma cells produce DKK1 to prevent osteoblast development (Zhang and Mager, 2019). These are just a few examples of how cancer and bone interact; readers are referred to Coleman et al. (2020) and Rao et al. (2020) and references therein for further details. The complex interplay between multiple organ systems in metastatic cancer means that almost all cancers have adverse effects on bone health (Drake, 2013).

## 4 Techniques for mathematical modeling of bone remodeling

Biochemical models of bone remodeling consider the population dynamics of bone cells, which are regulated by numerous chemical signaling factors. Temporal bone cell dynamics are modeled using ordinary differential equations (ODEs) (Tables 2–4), while spatiotemporal dynamics are modeled using partial differential equations (PDEs) and/or agent-based models (ABMs) (Table 1). ODEs can incorporate processes such as bone cell proliferation, differentiation, and death. Most bone remodeling ODEs are single-compartment models focusing on cells and signals locally within the bone microenvironment. ODEs can also describe multiple physiological compartments simultaneously to show how factors outside the bone microenvironment affect bone remodeling.

ODEs are the most common technique for mathematical modeling of bone remodeling but cannot explicitly include geometric and transport effects (Tables 2–4). Spatiotemporal models that incorporate these effects more accurately depict the bone remodeling process. For example, continuous PDEs can model the migration of osteoclasts and osteoblasts to specific locations within the remodeling site. These are important steps in bone remodeling that ODEs cannot resolve. However, PDEs are more computationally expensive to solve than ODEs because they include spatial and temporal effects. ABMs are less widely adopted for spatiotemporal modeling of bone remodeling (Araujo et al., 2014; Arias et al., 2018). Like PDEs, ABMs can model cell movement and how the spatial positioning influences the bone remodeling cycle. However, ABMs are discrete rather than continuous, so their computational intensity depends on the number of agents and the algorithms used to execute their interaction rules. In ABMs, cells are represented as agents that follow rules to move, proliferate, transform, die, and/or secrete signaling factors. The rules governing these cell actions consider the surrounding cell environment and probabilities for introducing stochasticity into the rules.

**FIGURE 5:**
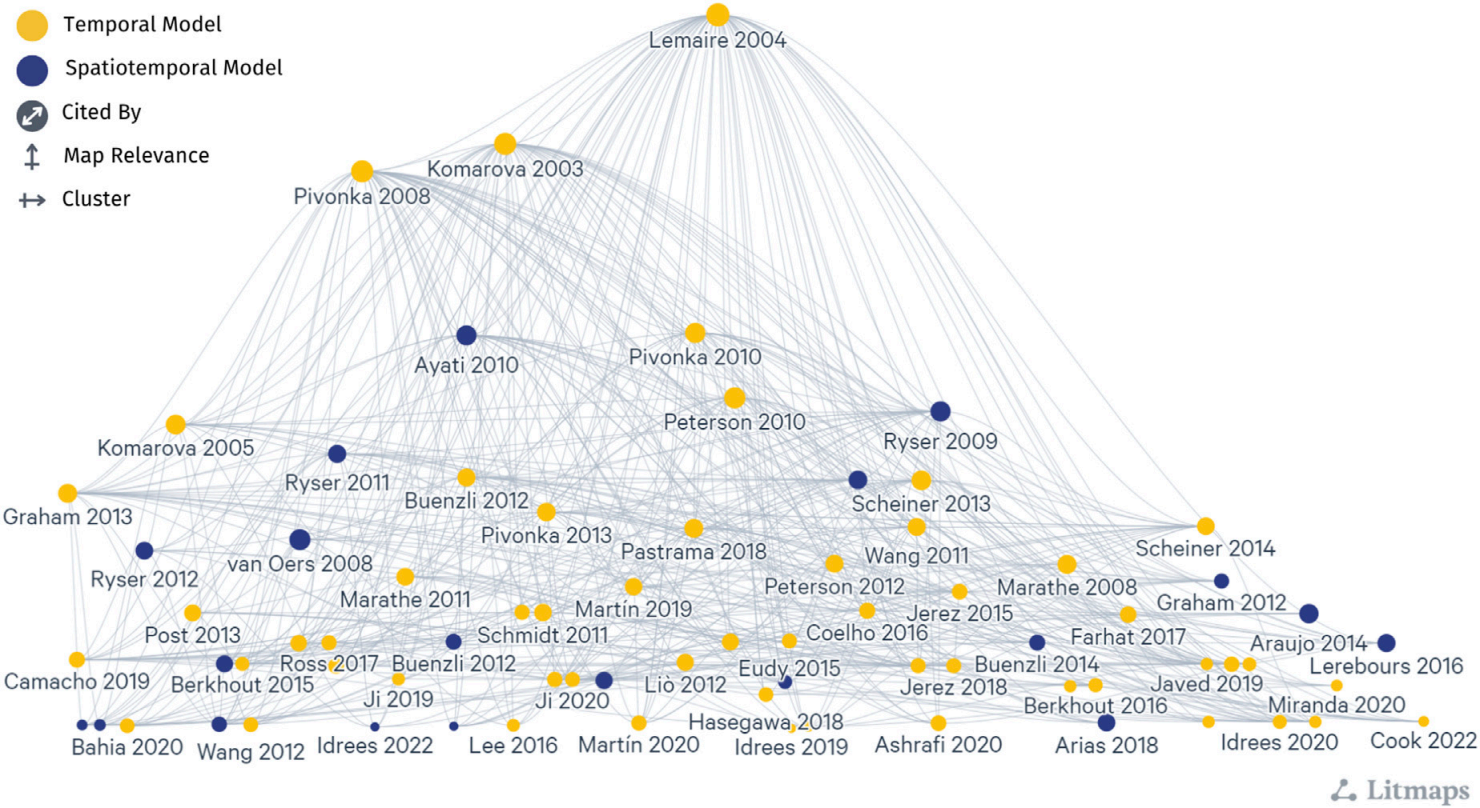
A network graph shows the citation relationship between mathematical models of bone remodeling that use the power law approach popularized by Komarova et al. (2003) and the mass action kinetics approach popularized by Lemaire et al. (2004) and Pivonka et al. (2008), which are detailed in Tables 1–3 and Supplementary Tables S1–S2. Each dot indicates a model publication, and curves represent a citation from one article to another. Yellow dots indicate temporal models, and dark blue dots indicate spatiotemporal models. Larger dots correspond to models with more publications (cited by). Models most connected to other articles are higher in the diagram (map relevance), while the left-to-right organization aids in clarity and label visibility (cluster). Note that not all labels are shown because of space constraints. The naming convention is the first author’s last name followed by the year of publication. This literature map was created using the online tool app.litmaps.com.

Two prevailing mathematical formulations that describe bone cell population dynamics and their biochemical signaling dynamics in the BMU are commonly incorporated into models of bone remodeling using ODEs, PDEs, and ABMs. One formulation is based on the power law approach, popularized for bone remodeling by Komarova et al. (2003). The second formulation uses the mass action kinetics as in the models of Lemaire et al. (2004) and Pivonka et al. (2008). These distinct approaches form the basis of many temporal and spatiotemporal models of bone remodeling (Figure 5, which includes all publications from Tables 1–3 and Supplementary Tables S1–S2 but not all labels are shown because of space constraints). Models that do not explicitly follow either approach (Kroll, 2000; Rattanakul et al., 2003; Martin and Buckland-Wright, 2004; Moroz et al., 2006; Moroz and Wimpenny, 2007; Akchurin et al., 2008; Ji et al., 2012; Proctor and Gartland, 2016; Chaiya and Rattanakul, 2017; Javed et al., 2018; Zhao and Zhang, 2019; Javed et al., 2020; Nutini et al., 2021; Jorg et al., 2022) (Table 4) are not included in Figure 5; however, most of those detailed in Supplementary Table S3 show citation connections for the field’s literature.

### 4.1 Power law approach

In biochemical models of bone remodeling, researchers represent the effects of signaling molecules on bone cell populations using different functional forms. The power law approach uses nonlinear functional relationships where output effects depend on an input raised to some power. These approximations are frequently used to model nonlinear biological systems because they capture complex dynamics relatively simply (Savageau, 1970; Vera et al., 2007; Srinath and Gunawan, 2010).

Models following the power law approach represent the lumped effects of types of signaling molecules on bone cell populations through the exponent terms in the power law functions. In the case of the Komarova et al. (2003) model, signaling molecules are grouped into general autocrine and paracrine signaling terms. The autocrine terms encompass all the signals released for self-regulation, e.g., osteoclast-derived signals that regulate the osteoclast population. The paracrine terms encompass all the signals other cells release, e.g., osteoblast-derived signals that regulate the osteoclast population. The general form for describing bone cell dynamics following the power law approach is

$$\frac{dA}{dt}=\alpha_{\text{A}}A^{g_{11}}B^{g_{21}}-\beta_{\text{A}}A$$ (1)

where A represents the number of cells of type A, B represents the number of cells of type B that interact with A through paracrine signaling, *g*_11_ represents autocrine (A to A) signaling action, *g*_21_ represents paracrine (B to A) signaling action, and *α*_A_ and *β*_A_ represent proliferation and degradation rate constants, respectively. Generally, *g*_*ij*_ denotes the combined effects of signals produced from cell type *i* (or a cascade involving this cell type) that regulate cell type *j*. Here, the proliferation of A (*j* = 1) depends on autocrine from A (*i* = 1) and paracrine from B (*i* = 2) signaling effects. The degradation rate is commonly assumed to be proportional to the current population.

The power law approach results in small parameter spaces. For example, the model in Komarova et al. (2003) contains only ten parameters fitted for a single BMU using experimental data from Parfitt (1994). A small parameter space requires fewer data for model calibration and validation and enables quick exploration of cell population balances through parameter sweeps. The lower computational complexity also allows researchers to connect the power law model to other biological system models, particularly for physiological homeostasis conditions. However, the empirical nature of power law models leads to ambiguity about which signaling factors control the bone remodeling cycle and how they interact mechanistically. The power law models cannot be easily extended for situations like diseases or treatments when these signals are perturbed outside the conditions used to fit the power law parameters. The lack of direct mechanistic interpretation is a common criticism of the power law approach (Moroz and Wimpenny, 2007).

### 4.2 Mass action kinetics approach

Another common form for ODE models of bone remodeling uses mass action kinetics. This fundamental concept is commonly used to model chemical and biological reactions, such as those seen in enzyme kinetics, ecological systems, and disease dynamics (Voit et al., 2015). In our classification, mass action kinetics includes Michaelis-Menten and Hill equations for enzyme and ligand binding kinetics. The mass action kinetics model structure for bone remodeling leverages the foundational model by Lemaire et al. (2004) and refinement by Pivonka et al. (2008) (Figure 5). The mass action kinetics approach is a major alternative to the power law approach as it better identifies how specific signaling factors affect the balance between osteoblast and osteoclast populations.

Bone models following the mass action kinetics approach capture the effects of signaling factors on cell dynamics with *π* terms. These terms represent the fraction of occupied receptors and are first defined by Lemaire et al. (2004). The model by Pivonka et al. (2008) simplifies the *π* terms using Hill functions defined in Eqs 3, 4 that represent ligand-receptor binding kinetics as activating or repressing processes, generalizing the work of Lemaire et al. (2004). Despite some differences between the *π* terms and models of Lemaire et al. (2004) and Pivonka et al. (2008), they share fundamental derivation steps. In the mass action kinetics approach, bone signaling factor actions are commonly represented by the reversible ligand-receptor relationship:

L+R↔L⋅R (2)

where *L* is the ligand, *R* is the receptor for the ligand, and *L* ⋅ *R* is the bound ligand-receptor complex. These ligand-receptor binding reactions in Eq. 2 for all ligands and receptors are converted into ODEs by applying mass action kinetics with the pseudo-steady state approximation. This assumes that the cellular response to signals is much slower than the dynamics of ligand-receptor binding. The *π* terms in Lemaire et al. (2004) are derived by finding the ratio of the ligand-receptor complex to the unbound ligand. Pivonka et al. (2008) generalizes these equations to obtain ligand concentrations for the formulaic *π* terms. Rather than deriving *π* terms from each ligand-receptor binding combination, Pivonka et al. (2008) assumes that Hill functions represent stimulation and inhibition of cell activity due to the presence of a signaling factor. Readers are referred to Lemaire et al. (2004) and Pivonka et al. (2008) for full derivation details. There are two forms of these Hill functions: one for activating signaling factors (Pivonka et al., 2008)

$$\pi_{\text{act},m}^{X}=\frac{X^{n}}{K_{1}+X^{n}}$$ (3)

and another for repressing signaling factors (Pivonka et al., 2008)

$$\pi_{\text{rep},m}^{Y}=\frac{1}{1+{\left(\frac{Y}{K_{2}}\right)}^{n}}$$ (4)

where *X* is the concentration of an activating signaling factor that affects cell type *m*, *K*_1_ is the activation coefficient, *n* is the Hill coefficient, *Y* is the concentration of a repressive signaling factor that affects cell type *m*, and *K*_2_ is the repression coefficient. Unlike enzyme kinetics, *K*_1_ and *K*_2_ are related to a cell response, not strictly biochemical dissociation constants. The concentrations of *X* and *Y* can be defined by ODEs or algebraic equations. It is important to note that a signaling factor can perform both activating and repressing actions and impact different cells, so it can have multiple corresponding *π* terms.

Although the *π* terms in Lemaire et al. (2004) and Pivonka et al. (2008) have slight derivation differences and biological assumptions, the resulting models are functionally similar. Consider a cell type A that is formed by the differentiation of precursor cells pA. This differentiation process is activated by signaling factor *X*_1_ and inhibited by signaling factor *Y*_1_. Apoptosis of A is activated by signaling factor *X*_2_ and inhibited by signaling factor *Y*_2_. Following the examples from (Pivonka et al., 2008), we provide the general form for describing bone cell dynamics following the mass action kinetics approach as

$$\frac{dA}{dt}=\alpha_{\text{pA}}pA\pi_{\text{act,pA}}^{X_{1}}\pi_{\text{rep,pA}}^{Y_{1}}-\beta_{\text{A}}A\pi_{\text{act,A}}^{X_{2}}\pi_{\text{rep,A}}^{Y_{2}}$$ (5)

where A is the population of cells of type A, pA is the population of precursor pA cells, $\pi_{\text{act,pA}}^{X_{1}}$ is the activation from signaling factor *X*_1_, $\pi_{\text{rep,pA}}^{Y_{1}}$ is the repression from signaling factor *Y*_1_, $\pi_{\text{act,A}}^{X_{2}}$ is the activation from signaling factor *X*_2_, $\pi_{\text{rep,A}}^{Y_{2}}$ is the repression from signaling factor *Y*_2_, and *α*_pA_ and *β*_A_ represent differentiation and apoptosis rate constants, respectively. The *π* terms replace the autocrine and paracrine exponents from Eq. 1 but still account for bone cells’ autocrine and paracrine signaling. Unlike the exponents in Eq. 1, the *π* terms allow the concentrations of signaling factors to depend on the population of BMU cells. Eq. 5 considers activating and repressing signals acting on both the source and sink terms. For different model scenarios considering various biological mechanisms, only one or neither of these signals may impact a term or more than one signal of the same type may be applied to a term.

The mass action kinetics approach results in larger parameter spaces than the power law approach. Whereas the power law model by Komarova et al. (2003) contains ten unknown parameters, the mass action kinetics models by Lemaire et al. (2004) and Pivonka et al. (2008) contain 23 and 30 parameters, respectively. The parameter increase is a consequence of the mechanistic incorporation of signaling factor effects. As a result, the mass action kinetics approach helps determine the importance of signaling factors within a specific study and how changes in their levels alter the bone remodeling cycle. The caveat of this approach is that more parameters can lead to overfitting of limited data. The mass action kinetics approach is also more computationally complex and expensive, limiting its use in larger multiscale models of biological systems.

### 4.3 Representative mathematical forms for modeling the BMU

In the following, we provide example mathematical forms for changes to bone volume due to remodeling and for bone cell population balances that are frequently considered in mathematical models for bone remodeling from a local perspective (i.e., focusing on the BMU).

#### 4.3.1 Bone volume

Regardless of the approach, models of bone remodeling generally include the dynamics of osteoblast and osteoclast cells. While cell populations’ evolution and signaling interactions vary between models, osteoblasts always form bone, and osteoclasts always break down bone. The net effect of bone regulation by these cells can generally be represented in ODE form by (Frost, 1964b)

$$\frac{dz}{dt}=R_{f}-R_{r}=k_{f}OBL-k_{r}OCL$$ (6)

where *z* is bone volume fraction, often corresponding to the ratio of bone volume to tissue volume (BV/TV) bone histomorphometric quantity (Bouxsein et al., 2010; Dempster et al., 2013), TV is frequently the trabecular volume (Burr and Akkus, 2014), *R*_*f*_ is the formation rate, *R*_*r*_ is the resorption rate, *k*_*f*_ is the formation rate constant for the action of the osteoblasts, and *k*_*r*_ is the resorption rate constant for the osteoclast activity. The variables OBL and OCL in Eq. 6 usually represent changes from the steady state population, sometimes called active cell populations. Additionally, bone volume may be replaced with bone mass or other relevant bone properties. Bone volume, total osteoblast population, and total osteoclast population cannot have negative values.

#### 4.3.2 Osteoclasts

A thorough understanding of osteoclast bone resorptive activity and population dynamics is crucial to predicting how much bone is resorbed during a remodeling cycle. The difference between the net formation and degradation terms determines the osteoclast population dynamics. The power law approach is used to mathematically represent these dynamics following Eq. 1 as (Komarova et al., 2003)

$$\frac{dOCL}{dt}=\alpha_{\text{OCL}}{OCL}^{g_{11}}{OBL}^{g_{21}}-\beta_{\text{OCL}}OCL$$ (7)

where OCL represents the number of osteoclasts, OBL represents the number of osteoblasts, *g*_11_ represents autocrine (osteoclast to osteoclast) signaling action, *g*_21_ represents paracrine (osteoblast to osteoclast) signaling action, and *α*_OCL_ and *β*_OCL_ represent proliferation and degradation rate constants, respectively. The proliferation of osteoclasts (*j* = 1) depends on autocrine from osteoclasts (*i* = 1) and paracrine from osteoblasts (*i* = 2) signaling effects. The degradation rate of osteoclasts is assumed to be proportional to the current population.

Some power law models modify the signaling dynamics to account for a specific molecular factor by reformulating the population dynamics and recalculating general signaling exponents (Komarova, 2005; Graham et al., 2013). For instance, if a signaling factor *F*_OCL_ alters osteoclast proliferation, the osteoclast equation is modified to become

$$\frac{dOCL}{dt}=F_{\text{OCL}}\alpha_{\text{OCL}}{OCL}^{g_{11}}{OBL}^{g_{21}}-\beta_{\text{OCL}}OCL$$ (8)

with new values for *g*_11_ and *g*_21_ as compared to Eq. 7.

By the mass action kinetics approach following Eq. 5, the population of osteoclasts is given by (Pivonka et al., 2008)

$$\frac{dOCL}{dt}=\alpha_{\text{pOCL}}pOCL\pi_{\text{act,pOCL}}^{X_{1}}-\beta_{\text{OCL}}OCL\pi_{\text{act,OCL}}^{X_{2}}$$ (9)

where OCL is the osteoclast population, pOCL is the preosteoclast population, $\pi_{\text{act,pOCL}}^{X_{1}}$ is the activation from signaling factor *X*_1_, $\pi_{\text{act,OCL}}^{X_{2}}$ is the activation from signaling factor *X*_2_, and *α*_pOCL_ and *β*_OCL_ represent differentiation and apoptosis rate constants, respectively. In Pivonka et al. (2008), differentiation and apoptosis terms are both activated and not inhibited. *X*_1_ is RANKL, and *X*_2_ is TGF-β. Different combinations of activating and repressing *π* terms are proposed in models from other publications. When following the mass action kinetics approach, researchers typically examine individual signaling factors during formulation. As a result, the overall structure of the osteoclast equation rarely undergoes drastic changes. Instead, new signaling factors are simply added through more *π* terms in the mass actions kinetics approach.

Uncommitted monocytes and preosteoclasts are rarely modeled as dynamic populations (thus, pOCL is a constant in Eq. 9). Osteoclasts are assumed to differentiate from a large pool of hematopoietic stem cells, so the uncommitted population is usually modeled as a fixed quantity. Although this assumption is reasonable for healthy bone remodeling, it loses validity when studying diseases where hematopoietic stem cell numbers are reduced (Weilbaecher, 2000; Matatall et al., 2016). Preosteoclasts are usually omitted for simplification under the assumption that remodeling is already occurring, i.e., the activation stage is assumed to occur instantaneously (Figure 1). However, this neglects the time needed to initiate this remodeling stage.

#### 4.3.3 Osteoblasts

Mathematical models must include osteoblast cell dynamics to understand changes in bone formation rates. Osteoblast population balances are similar to those of osteoclasts given in Eqs 7, 9, and these balances are modeled by the power law approach following Eq. 1 as (Komarova et al., 2003)

$$\frac{dOBL}{dt}=\alpha_{\text{OBL}}{OCL}^{g_{12}}{OBL}^{g_{22}}-\beta_{\text{OBL}}OBL$$ (10)

and by the mass action kinetics approach following Eq. 5 as (Pivonka et al., 2008)

$$\frac{dOBL}{dt}=\alpha_{\text{pOBL}}pOBL\pi_{\text{rep,pOBL}}^{Y_{1}}-\beta_{\text{OBL}}OBL$$ (11)

where the parameters here correspond to osteoblast (cell type *j* = 2) dynamics and pOBL is the preosteoblast population. In Pivonka et al. (2008), differentiation is inhibited, and apoptosis is neither activated nor inhibited. *Y*_1_ is TGF-β. Unlike osteoclasts, osteoblasts are modeled with one or two consumption terms. The use of one consumption term encapsulates osteoblast apoptosis and its conversion to other bone cells, such as osteocytes and bone lining cells. When models include two consumption terms, one tracks osteoblast conversion to osteocytes. The other consumption term tracks osteoblast apoptosis and other osteoblast cell losses.

Another difference between osteoblast and osteoclast population balances is that preosteoblast dynamics are commonly modeled. A study analyzing a generalized model of bone remodeling highlights the importance of preosteoblast populations (Zumsande et al., 2011). For instance, preosteoblasts release key signaling molecules that initiate the resorption phase of bone remodeling. Additionally, preosteoblast cell dynamics must be modeled because the number of osteoblasts is dictated by the preosteoblast population after proliferation (Buenzli et al., 2012b). Since osteoblasts do not proliferate, a decrease in bone formation may result from fewer preosteoblasts. Preosteoblast population balances follow the same form as those of osteoblasts and osteoclasts. The formation term represents differentiation from uncommitted osteoblasts, while the consumption term represents conversion to osteoblasts.

#### 4.3.4 Osteocytes

As research continues to indicate that osteocytes are essential coordinators of bone remodeling, it is vital to include their dynamics and populations in mathematical models. Osteocyte population balances are less commonly found in mathematical models than osteoclast and osteoblast balances but generally follow similar principles. In power law models, osteocytes are modeled following Eqs 8, 10 as (Graham et al., 2013; Cook et al., 2022)

$$\frac{dOCY}{dt}=F_{\text{OCY}}\alpha_{\text{OCY}}{OBL}^{g_{23}}$$ (12)

where OCY is the osteocyte population, *α*_OCY_ is the rate of conversion from osteoblasts, and *g*_23_ is osteoblast signals that influence the production of osteocytes (cell type *j* = 3) via osteoblast embedding. The factor *F*_OCY_ represents osteocyte signaling that activates and terminates the bone remodeling cycle.

Differently, models following the mass action kinetics approach base their osteocyte population on the change in bone volume as (Martin et al., 2019; Martin et al., 2020; Calvo-Gallego et al., 2023)

$$\frac{dOCY}{dt}=\eta \frac{dz}{dt}$$ (13)

where *η* is the average concentration of osteocytes embedded in the bone matrix and *z* is the bone volume fraction. Note the lack of degradation terms for long-lived osteocytes in Eqs 12, 13. Some disease or injury conditions may explicitly induce loss of osteocytes, which can be incorporated by including a loss term or by reducing the osteocyte initial condition (Graham et al., 2013; Cook et al., 2022).

## 5 Mathematical models of bone remodeling

**FIGURE 6:**
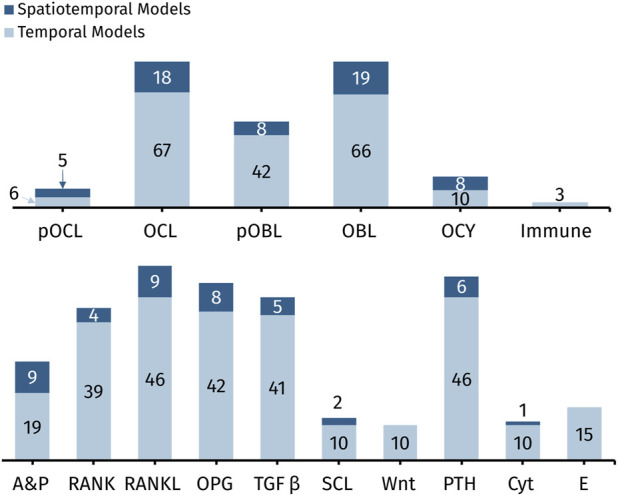
Quantitative comparison of cells (top row) and chemical signals (bottom row) commonly included in the 88 mathematical models of bone remodeling detailed in Tables 1–4. Abbreviations: pOCL, preosteoclasts; OCL, osteoclasts; pOBL, preosteoblasts; OBL, osteoblasts; OCY, osteocytes; Immune, immune cells; A&P, general autocrine and paracrine signaling; RANK, receptor activator of nuclear factor kappa-B; RANKL, receptor activator of nuclear factor kappa-B ligand; OPG, osteoprotegerin; TGF-β, transforming growth factor beta; SCL, sclerostin; Wnt, wingless-related integration site; PTH, parathyroid hormone; Cyt, cytokines other than RANK, RANKL, OPG, and TGF-β; E, estrogen.

Bone cells are typically represented similarly across spatiotemporal and temporal models. In the terminology adopted here, models that “include” a cell incorporate that cell as a state variable or dynamic variable, and “included” signals may be state variables, dynamic variables, constants, or implied. Although models following the power law approach imply several signaling molecules, this is indicated by their general autocrine and paracrine signaling representation, so only signaling features that are distinguished with a unique mathematical term are considered as “included” in the model. Figure 6 shows a quantitative comparison of the cells (top row) and chemical signals (bottom row) commonly included in the 88 mathematical models of bone remodeling detailed in Tables 1–4.

Osteoblast and osteoclast dynamics are included in almost every spatiotemporal and temporal model, whereas osteocyte dynamics are less commonly modeled (Figure 6; Tables 1–4). This is probably due to early assumptions about inert osteocytes, as described in Section 2.2. However, after osteocytes were found to play a mechanosensory role in bone remodeling, mathematical models began to include them when investigating mechanical effects on bone. For example, Moroz et al. (2006) is the earliest model with osteocytes, and the model includes mechanical stress. Moroz and Wimpenny (2007) introduces osteocyte regulation and, similar to Pivonka et al. (2008), defines autocrine and paracrine regulation mechanisms with more biologically accurate formulas, exploring four different receptor-ligand binding equations (Michaelis-Menten, Hill, Koshland-Nemethy-Filmer, and Monod-Wyman-Changeux) through stability analysis, ultimately concluding that the simpler Michaelis-Menten and Hill equations are most useful—consistent with models that adopt the mass action kinetics approach. Osteocytes are also typically included in mechano-chemo-biological models (Scheiner et al., 2013; Martin et al., 2019; Ashrafi et al., 2020; Calvo-Gallego et al., 2023). Other models with osteocytes aim to understand the effect of sclerostin, a product of osteocytes, on Wnt activation (Graham et al., 2013; Eudy et al., 2015; Cook et al., 2022). Although spatiotemporal and temporal models are remarkably similar in how often and which bone cells they explicitly model, they differ substantially in the number and combinations of signaling molecules modeled. Spatiotemporal models are discussed distinctly in Section 5.1.

The signaling molecules represented in ODEs for bone remodeling (Figure 6) differ partly due to the choice between the power law approach or the mass action kinetics approach. The power law approach uses general autocrine and paracrine signaling (Table 2). In contrast, the mass action kinetics approach explicitly models signaling interactions individually, such as RANK, RANKL, OPG, PTH, and TGF-β (Table 3). Furthermore, most power law models extend Komarova et al. (2003), and most mass action kinetics models extend Lemaire et al. (2004) or Pivonka et al. (2008) (Figure 5). Therefore, model extensions generally retain the signaling interactions of the original models. Sections 5.2, 5.3 address the evolution of signaling molecule representations since these foundational models. Table 2 and Supplementary Table S1 itemize the temporal mathematical models that follow the power law approach. Table 3 and Supplementary Table S2 focus on those that follow the mass action kinetics approach. Table 4 and Supplementary Table S3 include those temporal models that cannot be readily categorized as following either approach. Note that spatiotemporal models are also classified by approach in Supplementary Tables S1–S3.

### 5.1 Spatiotemporal models

The two most comprehensive spatiotemporal models are mechano-chemo-biological models that combine detailed biochemical and biomechanical processes (Table 1). One model uses a traditional transport-based approach that defines site-specific kinetic rate terms for each cell population equation (Lerebours et al., 2016). Another formulation uses a finite-element approach where each mesh point contains at most one BMU, and conditions are set to prevent the activation of bone formation or resorption in a BMU adjacent to another active BMU (Calvo-Gallego et al., 2023). Although both models include explicit parameters for RANK, RANKL, OPG, and TGF-β, these mechano-chemo-biological models have limited reuse for studying spatial variations for chemical interventions.

In contrast, only two non-biomechanical spatiotemporal models of bone remodeling explicitly model RANK-RANKL-OPG and TGF-β (Table 1). These models, Buenzli et al. (2011) and Buenzli et al. (2014), are 1D spatial extensions of the same temporal model. Buenzli et al. (2011) evaluates whether the biological pathways in Pivonka et al. (2008) are necessary and sufficient to capture the expected arrangement of cells in cortical bone and concludes that the model requires an additional differentiation stage for osteoclasts. Although this model includes more explicit parameters than other biochemical models, the values are not quantitatively compared to data. The Buenzli et al. (2011) model relies on theoretical simulation results and temporal study parameters and only estimates new parameters as needed. This is a common approach in spatiotemporal models, including those by Ayati et al. (2010) and Ryser and Murgas (2017). Arias et al. (2018) notes that there is no parameter fitting in their study and acknowledges that experimental data are necessary to quantify and validate the model. Yet, even though Ryser et al. (2010) calibrates a model with multiple datasets, the model is limited to fewer parameters and fewer explicit biological interactions. The authors of Ryser et al. (2010) offer the perspective that more parameters “compromise the balance between reliability and realism” by increasing the uncertainty of the model (Ryser et al., 2010).

Several spatiotemporal models focus on one phase of remodeling, such as osteoclast resorption (van Oers et al., 2008; Buenzli et al., 2012a; Arias et al., 2018), osteoblast formation (Taylor-King et al., 2020), or osteocyte dynamics (Ryser and Murgas, 2017). These models do not explicitly model multiple cell-cell or cell-signal interactions. Instead, they implicitly model the roles of RANK-RANKL-OPG, TGF-β, Wnt, and other signals using general autocrine and paracrine signaling parameters (Ryser and Murgas, 2017; Arias et al., 2018). In some cases, the models exclude the signaling mentioned above interactions in favor of more distinctive mechanisms and parameters. For instance, Buenzli et al. (2012a) includes parameters related to the involvement of blood vessels in osteoclast generation. Taylor-King et al. (2020), on the other hand, incorporates parameters for the shape and growth of osteocyte dendrites. Another example considers the energy-dependent dynamics of osteocytes (van Oers et al., 2008). The lack of explicit cell-cell and typical cell-signal interactions in these models may be attributed to their research motivations. The fewer bone cells and explicit signals seen in spatiotemporal models compared to temporal models (Figure 6) may also be due to their higher computational expense and complexity or to the lack of detailed spatial information for calibration and validation of such models at the cellular and molecular scales.

### 5.2 Power law models

The power law approach is discussed in Section 4.1, and its general application to the bone volume and cells of the BMU is shown in Section 4.3. Some adaptations based on Komarova et al. (2003) aim to explicitly capture signals that are only implicitly included in the original model, and others add new cells or signals (Table 2; Supplementary Table S1). In the latter group, Graham et al. (2013) adds state variables for osteocytes and preosteoblasts, along with implicit sclerostin/Wnt signaling terms. Cook et al. (2022) alters Graham et al. (2013) to explicitly account for systemic changes in Wnt-10b by using an enzyme kinetics approach to represent changes in Wnt-10b with a Hill function that modulates cell populations. Among the models that focus on explicitly capturing certain autocrine and paracrine signals is the spatial extension by Ryser et al. (2009). This model adds explicit state variables for RANKL and OPG by setting one of the original paracrine power parameters equal to zero, namely the one corresponding to osteoblast-derived osteoclast regulation, and formulating separate equations for RANKL and OPG levels. Camacho and Jerez (2021) follows Ryser et al. (2009) by dropping paracrine signal exponents to explicitly model TGF-β and Wnt as state variables in a temporal model. Camacho and Jerez (2021) also updates the cell population equations to incorporate TGF-β-induced osteoclast apoptosis and Wnt-induced osteoblast proliferation. In the bone metastasis model by Garzón-Alvarado (2012), tumor-induced changes in TGF-β and parathyroid hormone-related protein (PTHrP) are added as state variables.

### 5.3 Mass action kinetics models

The mass action kinetics approach is discussed in Section 4.2, and its general application to the bone volume and cells of the BMU is shown in Section 4.3. Extensions of Lemaire et al. (2004), aside from those based on Pivonka et al. (2008), integrate physiologically based models for calcium homeostasis (Peterson and Riggs, 2010; Peterson and Riggs, 2012; Ross et al., 2017) or integrate pharmacokinetics/pharmacodynamics (PKPD) to study the treatment of multiple myeloma (Marathe et al., 2008; Zhang and Mager, 2019) and osteoporosis (Marathe et al., 2011; Lemaire and Cox, 2019) (Table 3; Supplementary Table S2). So the signals and biological mechanisms added to models following Lemaire et al. (2004) focus on modifications necessary to capture disease dynamics or calcium homeostasis. For example, Peterson and Riggs (2010) adds equations for calcium across bone, plasma, kidneys, and gut. Their multi-compartment model consists of 28 ODEs incorporating molecules such as phosphate (PO_4_), non-bone PTH, calcitriol, and multiple intracellular osteoblast signals. Other models, such as Marathe et al. (2008), connect the PKPD models to the dynamics of bone remodeling through bone biomarkers that correlate with osteoclast activity. However, neither Marathe et al. (2008) nor Marathe et al. (2011) modify the bone dynamics to account for the onset of disease-related effects. Instead, the clinical data sets used for calibration and validation are from patients with the disease under study. The PKPD extension by Zhang and Mager (2019) amends the bone dynamics to account for the upregulation of DKK1 by multiple myeloma cancer cells. Two other models following Lemaire et al. (2004) consider Wnt-related signaling molecules. Eudy et al. (2015), based on Peterson and Riggs (2010), incorporates sclerostin effects and osteocyte activity in a PKPD model for the sclerostin antibody romosozumab. Lemaire and Cox (2019) also adds Wnt-related effects to study anti-sclerostin treatments for osteoporosis and derives a *π* term for sclerostin inhibition of the Wnt pathway based on mass action kinetics, assuming a constant Wnt concentration. While most models extend Lemaire et al. (2004), Schmidt et al. (2011) reduces the model to a system of two dependent variables (osteoblasts and osteoclasts) and uses dimensional analysis to determine important aspects of the model that control the dynamics. The reduction is achieved by changing the cell concentrations to vary with respect to the initial values given in Lemaire et al. (2004), casting the system into dimensionless variables, eliminating variables, and applying a quasi-steady-state approximation. The work also demonstrates negligible differences from the Lemaire et al. (2004) model for slow processes such as aging and estrogen deficiency. Like other models following Lemaire et al. (2004), the reduced model (Schmidt et al., 2011) is further extended to study postmenopausal osteoporosis and its treatment in other models including Post et al. (2013); Berkhout et al. (2015); Berkhout et al. (2016).

The biochemical ODEs derived from Pivonka et al. (2008) focus on adding mechanical or geometric effects, as well as PKPD models to study the treatment of osteoporosis and metastatic cancer-based bone diseases. Here, we highlight changes in bone cell dynamics and biochemical additions, but we forgo detailed descriptions of the mechanical and geometric model features (Table 3). Pivonka et al. (2013) modifies the bone population equations to incorporate specific surface-dependent geometric regulation effects. In Scheiner et al. (2013), TGF-β upregulation of progenitor cell differentiation is added, as well as mechanical strain-based regulation of preosteoblast proliferation and RANKL production of osteocytes. Scheiner et al. (2014) extends this model to study postmenopausal osteoporosis and its treatment with denosumab. In another extension of Scheiner et al. (2013), Martin et al. (2019) opts for a more biochemically focused model of osteocyte-driven mechanical regulation of bone remodeling. Osteocytes are added as a state variable proportional to the bone matrix fraction. A separate function accounts for nitric oxide (NO) production by osteocytes and co-regulation of RANKL by PTH and NO. For the Wnt/sclerostin-related effects of osteocytes, a multi-ligand Hill activator function is derived that assumes a constant Wnt concentration and equal binding affinities for DKK1 and sclerostin. Other models based on Pivonka et al. (2008) focus on disease and treatment. Wang et al. (2011) adds a state variable for multiple myeloma cancer cells and disease-specific regulatory mechanisms. Ji et al. (2014) extends the model to add VCAM1 regulation of preosteoblast and osteoblast cell populations and adds the role of small leucine-rich proteoglycans in multiple myeloma to study related treatments. Farhat et al. (2017) extends the Wang et al. (2011) prostate cancer model by adding the effects of calcium, active and latent TGF-β, and cancer-induced Wnt and DKK1. Trichilo et al. (2019) quantifies PTH administration under various conditions by combining features from several models, along with the intracellular osteoblast signaling equations in Peterson and Riggs (2010). Unlike most of the models discussed here, Trichilo et al. (2019) retains the state variable formulations for TGF-β, OPG, RANK, RANKL, OPG-RANKL binding, and RANK-RANKL binding to account for non-steady-state regulation during intermittent PTH administration. Additionally, the expression for preosteoblast expression of RANKL is modified to be more biologically accurate.

### 5.4 Systems biology models and discussion of opportunities for future models

Biochemical and cellular processes are the targets for most pharmaceutical and dietary interventions for bone diseases (Section 3). Considerable evidence from *in vivo* and *in vitro* studies have shown that prebiotics alter more than one aspect of the gut-bone axis (see Keirns et al. (2020) and references therein). Multifactorial aspects of the pathologies of bone loss in aging and menopause compounded with impacts of dietary factors on interactions between the immune, gastrointestinal, endocrine, and skeletal systems compel us to advocate for systems biology approaches to understand better this complex network of processes that connect dietary prebiotic and probiotic treatments to immune modulation and bone outcomes. Additionally, connecting the gut to bone through biological mechanisms is relevant more generally for orally administered bone therapeutics. Ultimately, bone loss is a systemic problem with multi-organ involvement. Improved mechanistic understanding of these complex relationships is needed to enhance interventions for bone loss. Multi-organ-system mathematical models of physiological and pathophysiological bone remodeling can help unravel these mechanisms while reducing the experimental costs associated with animal testing. There are few multi-compartment models of bone remodeling (Peterson and Riggs, 2010; Islam et al., 2021), and this approach warrants exploration in future models. Additional opportunities exist for creating multiscale models of bone remodeling by using ODEs, PDEs, and/or ABMs for interacting processes across temporal and spatial scales (Ford Versypt, 2021).

#### 5.4.1 Reversal cells and bone lining cells

Most mathematical models of bone remodeling have overlooked reversal cells and bone lining cells. Their absence is reasonable, given that these cells’ importance and mechanistic behavior are not well understood. Moreover, the modeling work of Arias et al. (2018) suggests that reversal and bone lining cells are not required to capture the dynamics of bone remodeling.

These cells are only included in one model each. Trichilo et al. (2019) models a constant population of bone lining cells. They also include a dynamic parameter that describes bone lining cell differentiation into osteoblasts. This parameter varies with PTH dosage, introducing another mechanistic avenue for PTH to regulate osteoblastogenesis. The mononuclear cells modeled in Martin and Buckland-Wright (2004) remove collagen fibrils from the bone surface during resorption. The behavior of these mononuclear cells is in line with current understanding of the role that reversal cells play in bone remodeling, indicating that the so-called mononuclear cells in Martin and Buckland-Wright (2004) are reversal cells. While reversal and bone lining cells have historically been excluded, future mathematical models paired with experimental work could help provide mechanistic insights into their functions.

#### 5.4.2 Cytokines

As mentioned earlier, Wnt plays an important role in modulating bone health. However, few models consider the details of this interaction. When mathematical models consider the Wnt/sclerostin interaction, Wnt levels are often excluded or assumed constant (Supplementary Tables S1–S3). Instead, these models focus on sclerostin levels (Figure 6) (Graham et al., 2013; Eudy et al., 2015; Lemaire and Cox, 2019; Martin et al., 2019). Two models with dynamic Wnt concentrations only allow Wnt to be altered through the presence of a tumor (Buenzli et al., 2012b; Farhat et al., 2017). Cook et al. (2022) includes a generalized dynamic concentration of Wnt-10b, where the amount of exogenous Wnt-10b (from dietary sources) influences BMU cell populations and bone volume.

While the RANK-RANKL-OPG and Wnt pathways are key regulators of the bone remodeling cycle, other cytokines modulate these signals and bone cell activity. Despite this, there is a distinct lack of variety in the cytokines considered in mathematical formulations of bone remodeling. Table 5 shows that only three cytokines aside from RANK-RANKL-OPG and TGF-β are explicted modeled: IL-6, MCSF, and IGF. One benefit of including other cytokines in bone models is the potential to explore their importance under various remodeling conditions, yet many models with cytokines lack this analysis. For example, IGF is included in Garzón-Alvarado (2012) to simulate osteosclerosis because tumors are known to increase IGF levels and thus increase osteoblast activity. However, this work does not directly analyze the effect of IGF on bone cell dynamics. Although the modeling and analysis of IL-6 and MCSF is limited, some studies analyze their role in remodeling using perturbation or sensitivity analysis.

Only two of the five models that include dynamic IL-6 levels analyze its effect on bone cell dynamics during the bone remodeling cycle (Table 5). IL-6 is included in Kroll (2000) in their study of the effects of PTH on bone dynamics, albeit in a simplified manner. Following Kroll (2000), Idrees et al. (2019) adapts Komarova et al. (2003) to include IL-6 in the simulation of intermittent versus continuous PTH treatment. Whereas Kroll (2000) scales theoretical parameter values so that osteoblast counts remain below 1000 cells, Idrees et al. (2019) performs a meta-analysis of various experiments to estimate parameter values statistically. This is an improvement over many other bone models that extract experimental values from multiple studies or rely on sparse and disparate clinical data sets.

The dynamics of IL-6 in PTH treatment models are simplistic compared to multiple myeloma models. Wang et al. (2011) accounts for IL-6 stimulation of multiple myeloma cells and IL-6 activation of RANKL. Wang et al. (2011) also performs a perturbation analysis to investigate the relative degree of RANKL activation by PTH versus IL-6 in homeostatic and diseased remodeling states and finds that PTH dominates over IL-6 under healthy bone remodeling conditions. However, IL-6 activates RANKL more than PTH under elevated IL-6 conditions, representing multiple myeloma disease. A limitation here is that the model lacks other cytokines and mechanisms that can alter RANKL activation, which may dominate over PTH and IL-6.

Wang et al. (2011) is extended by Ji et al. (2014) without any change in the representation of IL-6. However, the models differ in their parameter estimation and sensitivity analysis approaches. Wang et al. (2011) fits the model to achieve adult bone and cancer cell densities corresponding to literature values, while Ji et al. (2014) uses genetic algorithms. Although both studies include sensitivity analysis, Ji et al. (2014) performs sensitivity analysis at a fixed time point rather than over time as in Wang et al. (2011). Nonetheless, the results in Ji et al. (2014) support those in Wang et al. (2011). Upon varying 11 parameter values from 50% to 150%, Ji et al. (2014) shows that bone volume is most sensitive to TGF-β and the progression of multiple myeloma disease is most sensitive to IL-6. Given these results and the biological evidence that IL-6 contributes to bone pathophysiology, exploring its inclusion in future mathematical models of bone remodeling is pertinent.

The cytokine MCSF is a key activator of osteoclastogenesis, yet analysis of its effects via mathematical models of bone remodeling is more sparse than IL-6. Martin and Buckland-Wright (2004) is the first model that includes the role of MCSF. This model investigates the depth and duration of osteoclast erosion during resorption and models MCSF as a scalar variable but assumes it is always present. A sensitivity analysis of resorption depth indicates that changes in MCSF levels are equivalent to changes in maximum osteoclast activity, and both effects are minor compared to TGF-β. Lerebours et al. (2016) and Pivonka et al. (2013) are biomechanical models that include MCSF activation of uncommitted osteoclasts. However, they assume the MCSF concentration is constant, resulting in a continuous activator function term.

Proctor and Gartland (2016) investigates the kinetics of MCSF via network-based ODEs. As with Lerebours et al. (2016) and Pivonka et al. (2013), this work investigates the effect of mechanical loading and the effects of PTH and circadian rhythm. In contrast, the network analysis includes multiple parameters that capture the role of MCSF in remodeling (outlined in Table 5). Sensitivity analysis of the model shows that the secretion rate of MCSF by osteoblasts and preosteoclasts and the MCSF degradation rate result in a change of more than 5% in bone mass. When the rate of degradation doubles, bone mass increases by more than 60%. One limitation of this study is the assumption that MCSF secretion rates are considered equal across cell types. Still, these results warrant further mathematical investigation of MCSF in homeostatic and pathological bone remodeling. Ultimately, future mathematical models of bone biology should explore the complex and coordinated role of cytokines, growth factors, and hormones in bone remodeling.

#### 5.4.3 Immune cells

Despite the established interest in osteoimmunology, few bone models include immune cells (Table 6). Most bone models that investigate the role of immune cells do so in the context of bone injury and repair (Trejo et al., 2019; Tourolle et al., 2021; Baratchart et al., 2022). Of particular interest is the model by Baratchart et al. (2022). Hypothesis testing of candidate models determines the interaction of monocytes, macrophages, injury factors, and inflammatory factors in bone cell dynamics. The model is supported by biological data, with the parameters calibrated with one set of experimental data and validated with another. These methods show how researchers can elucidate the mechanisms of complex bone-immune dynamics using mathematical models. However, bone injury and repair models describe the acute healing process of fractures, which has different signaling pathways than the continuous bone renewal or remodeling process for homeostasis and skeletal integrity over a lifetime. Therefore, bone healing models cannot be directly applied to the bone remodeling processes.

The few mathematical models of bone remodeling that incorporate immune cells are outlined in Table 6. Although the models by Akchurin et al. (2008) and Proctor and Gartland (2016) seemingly include immune cells (mononuclear cells and hematopoietic stem cells), these are simply different osteoclast progenitors. As mentioned earlier, these cells are often lumped into a general class of uncommitted osteoclasts or preosteoclasts.

Of the models listed in Table 6, only Islam et al. (2021) investigates the dynamic effect of multiple immune cells in bone remodeling. The work includes a three-compartment physiologically based PK model for differentiating naïve CD4^+^ T cells into Treg cells in the gut, blood, and bone. These Tregs then influence TGF-β production in the bone and induce Wnt-10b production. The physiologically based PK model is then linked to a bone remodeling model that includes the local effects of systemic changes in Wnt-10b (Cook et al., 2022). Since this is the only mathematical description of nonlocal immune effects on bone dynamics, significant opportunities remain for future research to explore multi-organ systemic interactions between the skeletal and immune systems.

#### 5.4.4 Endocrine system and pharmaceuticals

Despite its documented importance in bone remodeling and estrogen-deficient osteoporosis, the incorporation of dynamic estrogen levels in mathematical models of bone remodeling is underwhelming (Table 7). Analysis of the 88 cell population-based bone modeling publications in Tables 1–4 reveals that roughly a third (30 of 88) mention or model estrogen. Of these 30 models, half (15) capture estrogen effects in their mathematical model, while the other half only mention estrogen briefly. Most models that mention estrogen cite evidence that estrogen deficiency is involved in osteoporosis or modulates bone remodeling (Kroll, 2000; Marathe et al., 2008; Pivonka et al., 2008; Marathe et al., 2011; Garzón-Alvarado, 2012; Ross et al., 2012; Buenzli, 2015; Chen-Charpentier and Diakite, 2016; Lee and Okos, 2016; Jerez et al., 2018; Bahia et al., 2020; Javed et al., 2020). The remaining articles that mention estrogen acknowledge that estrogen is not incorporated into their model or that integration of estrogen is an opportunity for future models (Peterson and Riggs, 2010; Buenzli et al., 2012b; Coelho et al., 2016; Martin et al., 2019). Altogether, this indicates that only about 15% of all bone remodeling models mathematically account for estrogen-induced biochemical changes in bone cell dynamics.

All models with estrogen effects are osteoporosis-specific models, and their respective mathematical representations are outlined in Table 7. Several models do not consider dynamic estrogen levels (Lemaire et al., 2004; Scheiner et al., 2013; Lemaire and Cox, 2019; Lemaire and Cox, 2019; Martin et al., 2019; Trichilo et al., 2019; Larcher and Scheiner, 2021). Instead, they model the effects of estrogen deficiency by altering RANKL, OPG, PTH, and TGF-β. Lemaire et al. (2004) and Lemaire and Cox (2019) manually lower the OPG and TGF-β production parameters for osteoporotic scenarios. Trichilo et al. (2019) and Martin et al. (2019) increase RANKL levels with a RANKL dosage term fitted to OVX rat data and clinical postmenopause data. Scheiner et al. (2014) accounts for more estrogen-deficiency effect by using *π* terms to capture disease-related increases in RANKL, decreases in mechanical loading sensitivity, and denosumab competition with RANK and OPG.

Two models with dynamic estrogen levels aim to determine the most effective therapeutic dose to prevent bone loss. In Rattanakul et al. (2003), periodic estrogen treatment is modeled with a linear increase in osteoclast removal. Chaiya and Rattanakul (2017) reformulates the model to be explicitly piecewise. Using the power law approach to illustrate, the osteoclast rate equation becomes

$$\frac{dOCL}{dt}=\left\{\begin{array}{cc} \alpha {OCL}^{g_{11}}{OBL}^{g_{21}}-\beta OCL\; & \text{if }t\neq nT\\ \alpha {OCL}^{g_{11}}{OBL}^{g_{21}}-\beta OCL-\rho OCL\; & \text{if }t=nT \end{array}\right.$$ (14)

where *ρ* is the parameter related to estrogen treatment, *T* is the prescribed dose time, and *n* is the treatment number. In addition to Eq. 14, Chaiya and Rattanakul (2017) adds a constant term to capture the osteoblast-stimulating effects of estrogen treatment on the osteoblast rate equation. The motivation of this work is to understand alternative treatment regimes for ERT because long-term continuous treatment, while effective in increasing bone volume, has been shown in some studies to increase the risk for breast cancer and heart disease (Levin et al., 2018).

Models of dynamic estrogen loss often use an exponential decay equation for estrogen concentration or an estrogen-dependent dynamic parameter (Schmidt et al., 2011; Post et al., 2013; Berkhout et al., 2015; Berkhout et al., 2016). Although Berkhout et al. (2015) notes that estrogen concentration could better explain disease dynamics, they opt for the decay equation instead due to the high uncertainty in their model. In addition to estrogen decay, Schmidt et al. (2011) and Post et al. (2013) modify the OPG production parameter following Lemaire et al. (2004). Javed et al. (2018) derives an alternate formulation for remodeling altogether to simplify the mass action kinetics models proposed by Lemaire et al. (2004) and Pivonka et al. (2008). Estrogen changes are represented by a hyperbolically scaled estrogen term that modulates the RANKL state variable. In contrast, Jorg et al. (2022) models estrogen as an age-dependent concentration with a characteristic time scale of menopause onset that is fit to clinical data. This model also considers how estrogen alters sclerostin levels.

Most models of estrogen dynamics only consider bone cell or RANK-RANKL-OPG interactions, despite accumulating evidence that estrogen modulates other mechanisms of bone remodeling such as Wnt signaling and immune-bone interactions (see Section 3.2.2). Future models need to incorporate these complex dynamics in the ongoing effort to improve mechanistic understanding of estrogen-deficient osteoporosis.

Many bone remodeling models explore the effects of one or more drugs on bone health (Riggs and Cremers, 2019; Ait Oumghar et al., 2020) (Supplementary Tables S1–S3). Researchers typically start by modeling a healthy or diseased remodeling cycle (or leveraging existing models) and then extend the process to include drug effects. For example, glucocorticoid therapies and their interactions with the bone remodeling cycle are modeled in Lemaire et al. (2004), Schmidt et al. (2011), and Lemaire and Cox (2019). These models alter one parameter related to a symptom of glucocorticoid treatment, specifically reduced osteoblast populations. This essentially involves reducing *α*_OBL_ in Eq. 10 or Eq. 11. However, reducing one parameter corresponding to an effect observed with glucocorticoid treatment is a simplistic approach that may miss important mechanistic impacts on the bone remodeling cycle.

Other models (e.g., Jorg et al. (2022)) study bisphosphonates, denosumab, or romosozumab. The antiresorptive drugs are modeled by combining the PKPD information of the drug of interest and an already-established mathematical model of the BMU. PK information consists of factors that explain how the drug disperses in the body. These are usually differential equations that track the amount of a drug in a target area. PD information describes how the drug interacts with the body. The effects can be shown directly through new parameters in the model or implicitly applied by changing an existing parameter.

#### 5.4.5 Gut metabolites and immune connections

Whereas the immune-bone connection gained traction in the 2000s, the link between gut and bone metabolism is more recent. So it follows that fewer mathematical models of bone remodeling consider gut-mediated impacts on bone health. Only one mathematical model of bone remodeling incorporates gut and immune cells (Islam et al., 2021). This model explores butyrate treatment of bone through T-cell-mediated changes in Wnt-10b. Although much is still unknown about the gut-bone connection, the Islam et al. (2021) model is initialized with data from mouse experiments that complement the mathematical model. Sensitivity analysis and *in silico* hypothesis generation link the calculated parameters to experimental conditions that can be modified to explore new treatments. This highlights the benefit of experimentally supported mathematical models of bone remodeling. The multi-compartment modeling approach of Islam et al. (2021) and Peterson and Riggs (2010) provide examples of how mathematical models of bone remodeling may explore relationships of systemic multi-organ effects.

#### 5.4.6 Metastatic cancer cells

Similar to pharmaceutical modeling, most cancer models start by modeling normal bone homeostasis and supplement it with an equation for tumor dynamics (Marathe et al., 2008; Ayati et al., 2010; Araujo et al., 2014; Ji et al., 2014; Coelho et al., 2016; Farhat et al., 2017). Many cancer models also add or adjust parameters such as RANKL, TGF-β, and PTH, which are known to be modified by tumors. The populations of these cancer tumor cells (T) are usually modeled in one of two ways. The first modeling method is based on growth curves, as in

$$\frac{dT}{dt}=\gamma Tdensity\lambda -\eta T$$ (15)

where *γ* and *η* are growth and decay parameters, Tdensity is a relationship between the current and maximum cancer cell population, and *λ* is an additional relationship term to capture the effects of other cancer interactions considered (Ayati et al., 2010; Buenzli et al., 2012b; Coelho et al., 2016; Zhang and Mager, 2019; Miranda et al., 2020). For example, in the Coelho et al. (2016) model, *λ* in Eq. 15 corresponds to the concentration of osteoclasts.

The second common way to model cancer populations follows the mass action kinetics approach. Here, the populations are controlled by different signaling factors represented by *π* terms (Wang et al., 2011; Ji et al., 2014). In a publication that uses both modeling methods, the growth curve is better for early cancer, and the mass action kinetics structure is better for established cancer (Farhat et al., 2017).

The mechanisms of tumor growth and metastasis have important spatial considerations. This is the primary motivation behind existing bone remodeling PDEs and ABMs of cancer (Ayati et al., 2010; Ryser et al., 2012; Araujo et al., 2014), which track the movement of cancer cells in space.

## 6 Conclusion

Understanding the controlling factors in bone remodeling is vital for treating bone-related diseases. Existing mathematical models of remodeling have provided valuable insight into the mechanisms of remodeling. However, the scattered and varied parameter fitting techniques are a common limitation across these models. It is essential to calibrate and validate the models with more robust datasets through collaborations or rigorous collation of existing data, e.g., Ledoux et al. (2022), to develop biologically accurate and reusable bone models. With the emergence of new technologies for measuring single-cell and spatially resolved ’omics and for *in vivo* dynamic imaging modalities, static and dynamic data at the tissue, cellular, and molecular scales should be leveraged increasingly to enhance modeling efforts for bone remodeling. As modeling grows in popularity, many more insights will be drawn from mathematical models, such as the ones discussed in this review. Systems biology is needed to meet the challenges associated with viewing bone remodeling as a systemically controlled process in health and disease.
