# Supplementary material for: A review of mathematical modeling of bone remodeling from a systems biology perspective
Source: Front Syst Biol. 2024 Apr 9;4:1368555. doi: 10.3389/fsysb.2024.1368555 (PMC11864782; doi:10.3389/fsysb.2024.1368555)
Supplement: Supplementary file 1 [file DataSheet1.PDF]

A review of mathematical modeling of bone remodeling from a systems biology perspective

Carley V. Cook [1,†], Ariel M. Lighty [1,†], Brenda J. Smith [2,3], and Ashlee N. Ford Versypt [1,4,5,*]
[1]Department of Chemical and Biological Engineering, University at Buffalo, The State University of New York, Buffalo, NY, USA
[2]Indiana Center for Musculoskeletal Health, School of Medicine, Indiana University, Indianapolis, IN, USA
[3]Department of Obstetrics and Gynecology, School of Medicine, Indiana University, Indianapolis, IN, USA
[4]Department of Biomedical Engineering, University at Buffalo, The State University of New York, Buffalo, NY, USA
[5]Institute for Artificial Intelligence and Data Science, University at Buffalo, The State University of New York, Buffalo, NY, USA
[†]These authors contributed equally to this work and share first authorship.
*Correspondence:
Ashlee N. Ford Versypt
ashleefv@buffalo.edu

SUPPLEMENTARY MATERIAL

1 Supplementary tables

Tables S1–S3 provide summary descriptions of the mathematical models of bone remodeling reviewed in the manuscript. Table S1 considers those that follow the power law approach. Table S2 considers those that follow the mass action kinetics approach. Table S3 considers those that do not follow the power law or mass action kinetics approaches used in the models in Tables S1 and S2.

Table S1: Mathematical models of bone remodeling that follow the power law approach. All models in the table include osteoblasts (OBL) and osteoclasts (OCL), except for Ryser et al. (2012) which only includes OCL, so these are not explicitly listed in the table. All the models also include the general autocrine and paracrine signals. † denotes that a model has a stochastic aspect. ^{d} denotes that a model is a system of delay differential equations (DDEs). Abbreviations: basic/bone multicellular unit (BMU), calcitonin (CT), insulin-like growth factor (IGF), interleukin-6 (IL-6), metastatic tumor (MT), multiple myeloma (MM), ordinary differential equations (ODEs), osteocytes (OCY), osteoprotegerin (OPG), parathyroid hormone-related protein (PTHrP), partial differential equations (PDEs), preosteoblasts (pOBL), preosteoclasts (pOCL), prostate cancer (PC), receptor activator of nuclear factor kappa-B (RANK), receptor activator of nuclear factor kappa-B ligand (RANKL), sclerostin (SCL), transforming growth factor beta (TGF-β), wingless-related integration site (Wnt).

**Model**	**Cells**	**Signaling Mechanisms**	**Treatments**	**Motivation/Insights**
**ODEs**				
Komarova et al. (2003)	-	-	-	1. Models the different dynamics of remodeling<br>2. Power law application to autocrine/paracrine signals<br>3. Simplified model shows complex dynamics
Komarova (2005)	-	-	PTH	1. Models the effect of PTH administration on bone cells<br>2. Points to the importance of correct dosing with PTH
Garzón-Alvarado (2012)	MT	IGF, PTHrP, TGF-β	-	1. Models two different MT types<br>2. Qualitative view of MT dynamics in bone
Liò et al. (2012)	Bacteria	RANKL	-	1. Develops a model for osteomyelitis<br>2. Model shows how bacteria population in osteomyelitis affects bone
Graham et al. (2013)	pOBL, OCY	Implied SCL	-	1. Focuses on the role of OCY in remodeling<br>2. Qualitative exploration of OCY and SCL as therapeutic targets
Jerez and Chen (2015)	-	Generalized external influence	-	1. Identifies areas of stability for Komarova et al. (2003)<br>2. Adds new OCL influencer to account for external inputs
Chen-Charpentier and Diakite (2016)	-	-	-	1. Adds a delay aspect to Komarova et al. (2003) to capture the signaling time<br>2. Explores the differences in dynamics of Komarova et al. (2003) and the delay model
Coelho et al. (2016)	pOBL, pOCL, MT	PTH	Anti-cancer, bisphosphonates, denosumab	1. Focuses on how PTH and other therapies interact with a bone metastatic environment<br>2. Provides insight on the possibility of anti-resorptive treatments to prevent tumor growth

Table S1 continued from previous page

Model	Cells	Signaling Mechanisms	Treatments	Motivation/Insights
Jerez et al. (2018)[†]	-	Environmental randomness	-	1. Focuses on realistic variations in bone remodeling<br>2. Stochastic model shows a fluctuating periodic solution
Camacho and Jerez (2019)	MT	-	Denosumab, radiotherapy	1. Develops an optimal control model of MT in bone<br>2. Explores different treatment effectiveness for MT
Idrees et al. (2019)[d]	pOBL	IL-6, PTH	PTH	1. Combines Komarova et al. (2003) and Kroll (2000) into a DDEs model of PTH<br>2. Indicates that a larger dose of PTH is more effective
Javed et al. (2019)	-	Aging factor, RANKL	-	1. Focuses on the dynamics of bone remodeling through analysis of a bone remodeling model<br>2. Produces a stability analysis of bone remodeling
Idrees and Sohail (2020)	-	CT	CT	1. Explores the effects of dosed CT on bone remodeling<br>2. Points to the need to have intermittent dosing of CT
Miranda et al. (2020)	MT	-	Bisphosphonates, denosumab, paclitaxel, proteasome inhibitors	1. Explores the effects of several drugs on bone remodeling in the presence of an MT<br>2. Model considers drug resistance after prolonged treatment
Camacho and Jerez (2021)	MT	TGF-β, Wnt	Biphosphonates, chemotherapy	1. Directly models TGF-β and Wnt by removing OBL paracrine exponent<br>2. Explores treatment of MT with varying levels of TGF-β and Wnt
Islam et al. (2021)	pOBL, OCY	Wnt-10b, implied SCL	Butyrate, Wnt-10b	1. Determines if butyrate activation of Tregs accounts for bone volume increase seen from butyrate studies<br>2. Points to other influences of butyrate on bone remodeling
Cook et al. (2022)	pOBL, OCY	Wnt-10b, implied SCL	-	1. Explores the effect of Wnt-10b alterations on bone remodeling<br>2. Points to a previously unidentified indirect relation of Wnt-10b to OCL
**PDEs**				
Ryser et al. (2009)	-	OPG, RANKL	-	1. Focuses on the spatiotemporal dynamics of a BMU<br>2. Model predicts the movement of a BMU through bone

Table S1 continued from previous page

Model	Cells	Signaling Mechanisms	Treatments	Motivation/Insights
Ayati et al. (2010)	MM	-	Parameters related to proteasome inhibitors	1. Focuses on the movement of cells and MM interactions<br>2. Spatial model of MM and bone allows for the exploration of treatment targets
Ryser et al. (2010)	-	OPG, RANKL	-	1. Captures the effects of RANKL and OPG fields on bone remodeling<br>2. Model explains how OBL and OCL communicate without direct contact
Graham and Ayati (2012)	-	-	-	1. Considers the geometrical aspects of bone remodeling<br>2. Produces a PDE version of Komarova et al. (2003) using a level set approach
Ryser et al. (2012)	pOCL, MT	Only autocrine, OPG, PTHrP, RANKL	-	1. Captures the effects of MT with fields of RANKL, OPG, and PTHrP<br>2. Points to different responses to varying levels of OPG
Ryser and Murgas (2017)	OCY	-	-	1. Applies evolutionary game theory to a spatial model that includes OCY<br>2. Highlights the impact of geometry on bone remodeling
Peyroteo et al. (2019)	-	-	-	1. Converts Komarova et al. (2003) into a spatiotemporal model<br>2. Compares the results of three different numerical techniques used to solve the same equation set
Baldonedo et al. (2021)	pOBL, OCY	Implied SCL	-	1. Extends into a spatiotemporal model<br>2. Compares two-dimensional model to original results of Graham et al. (2013)
Idrees and Sohail (2023)	MM	CT, PTH	Bisphosphonates, PTH, anti-cancer	1. Extends Ayati et al. (2010) to include PTH and CT<br>2. Shows the effects of select hormones and treatments in a cancer environment

Table S2: Mathematical models of bone remodeling that follow the mass action kinetics approach. All models in the table include osteoblasts (OBL), osteoclasts (OCL), osteoprotegerin (OPG), parathyroid hormone (PTH), receptor activator of nuclear factor kappa-B ligand (RANKL), and transforming growth factor beta (TGF-β) so these are not explicitly listed in the table. * denotes that a model has a biomechanical aspect. Abbreviations: basic/bone multicellular unit (BMU), calcium (Ca), dickkopf-related protein 1 (DKK1), insulin-like growth factor 1 (IGF-1), interleukin-6 (IL-6), macrophage colony-stimulating factor (MCSF), multiple myeloma (MM), nitric oxide (NO), N-telopeptide (NTX), ordinary differential equations (ODEs), osteocytes (OCY), parathyroid hormone-related protein (PTHrP), partial differential equations (PDEs), phosphate (PO_4), preosteoblasts (pOBL), preosteoclasts (pOCL), prostate cancer (PC), pharmacokinetic (PK), pharmacodynamic (PD), prostate-specific antigen (PSA), pulsed electromagnetic field (PEMF), small leucine-rich proteoglycans (SLRPs), sclerostin (SCL), semaphorin-3A (Sema3A), urokinase plasminogen activator receptor-associated protein (Endo180), vascular cell adhesion molecule 1 (VCAM1), very late antigen-4 (VLA4), wingless-related integration site (Wnt).

Model	Cells	Signaling Mechanisms	Treatments	Motivation/Insights
**ODEs**				
Lemaire et al. (2004)	pOBL	-	Generic anti-resorptive, generic bone formation	1. Mechanistic model of bone remodeling explores diseased states<br>2. Points to formation therapies that increase the amount of pOBL
Pivonka et al. (2008)	pOBL	-	-	1. Focuses on the implications of the RANKL/OPG relationship<br>2. Updates the receptor-ligand relationship presented by Lemaire et al. (2004)<br>3. Model is most responsive when pOBLs express RANKL and OPG is expressed by OBLs
Marathe et al. (2008)	pOBL	-	Denosumab	1. Develops a model that shows bone remodeling response to denosumab in MM patients<br>2. Model connects denosumab response through experimentally measurable NTX
Peterson and Riggs (2010)	pOBL	Ca, PO_4	PTH	1. Focuses on the systemic factors in bone remodeling and Ca homeostasis<br>2. Multicompartment model connects to bone, gut, kidneys, and parathyroid
Pivonka et al. (2010)	pOBL	-	-	1. Determines if Pivonka et al. (2008) can represent diseases related to RANKL/OPG<br>2. Points to dual therapies being best at treating the represented bone diseases

Table S2 continued from previous page

Model	Cells	Signaling Mechanisms	Treatments	Motivation/Insights
Marathe et al. (2011)	pOBL	-	Denosumab, ibandronate	1. Characterizes the action of denosumab and ibandronate<br>2. Explores the action of these drugs on osteoporotic bone remodeling
Schmidt et al. (2011)	-	-	Estrogen, glucocorticoids, vitamin D	1. Reduces Lemaire et al. (2004) to a smaller but dynamically similar system<br>2. Shows that, on the timescale of disease progression modeling, OBL and OCL are sufficient for bone remodeling dynamics
Wang et al. (2011)	pOBL, MM	IL-6, VCAM1, VLA4	-	1. Focuses on the MM feedback mechanisms resulting in bone lesions<br>2. Points to only needing two feedback mechanisms for MM to progress
Buenzli et al. (2012b)	pOBL, PC	PSA, PTHrP, Wnt	-	1. Captures OBL production through pOBL proliferation<br>2. Model shows how pOBL proliferation could be affected by signals such as Wnt and PTH
Peterson and Riggs (2012)	pOBL	Ca, PO_4	Denosumab	1. Extends Peterson and Riggs (2010) to include denosumab treatment<br>2. Explores the effect of start-stop-restart treatment regimens
Ross et al. (2012)	pOBL	-	PTH	1. Adds PTH action on osteoblasts to Lemaire et al. (2004)<br>2. Model exhibits the catabolic and anabolic behavior of PTH
Wang and Qin (2012)	pOBL	-	PEMF	1. Extends Pivonka et al. (2008) to respond to PEMFs<br>2. Identifies parameter sets that represent the physiological response to PEMF
Scheiner et al. (2013)*	pOBL	-	-	1. Combines bone cell populations and mechanical strains into one model<br>2. Model depicts bone loss over time
Pivonka et al. (2013)*	pOBL, pOCL	MCSF (assumed constant)	-	1. Focuses on the impact of bone microstructure density<br>2. A mechano-chemo-biological model links to the geometrical structure of bone
Post et al. (2013)	-	Estrogen	Ca placebo, tibolone	1. Compares clinical data of osteoporosis to model (Schmidt et al., 2011)<br>2. Identifies two types of responders to Tibolone

Table S2 continued from previous page

Model	Cells	Signaling Mechanisms	Treatments	Motivation/Insights
Ji et al. (2014)	pOBL, MM	IL-6, SLRPs, VCAM1, VLA4	-	1. Focuses on MM dynamics with bone remodeling<br>2. Simulations of tumor removal show why bone rarely heals
Scheiner et al. (2014)*	pOBL	-	Denosumab	1. Alters Scheiner et al. (2013) to a postmenopausal state<br>2. Explores the effect of denosumab on diseased state
Berkhout et al. (2015)	-	Estrogen	Ca placebo	1. Applies a reduced model (Schmidt et al., 2011) to postmenopausal populations<br>2. Demonstrates that the model is relevant for multiple placebo populations
Lee and Okos (2016)	pOBL	Ca, IGF-1	-	1. Adds IGF-1 to Pivonka et al. (2010)<br>2. Explores how different perturbations to the parameters affect bone remodeling
Eudy et al. (2015)	pOBL, OCY	Ca, PO_4, SCL, Wnt (assumed constant)	Romosozumab	1. Explores the effects of OCY-produced SCL on the remodeling cycle<br>2. Alteres Peterson and Riggs (2010) to show how SCL and SCL-blockers interact with bone remodeling
Berkhout et al. (2016)	-	Estrogen	Alendronate (bisphosphonate)	1. Focuses on the long-term use of alendronate<br>2. Utilizes Schmidt et al. (2011) and adds PK/PD information for alendronate
Farhat et al. (2017)	pOBL, PC	Ca, DKK1, PSA, PTHrP, Wnt	-	1. Focuses on how PC interrupts the bone remodeling balance<br>2. Blocking Wnt with DKK1 might prevent tumor growth
Ross et al. (2017)	pOBL	Ca, PO_4	Bisphosphonate, generic RANKL inhibitor	1. Combines several models of bone remodeling to get a multicompartment model<br>2. Explores the action of anabolic and antiresorptive compounds on bone
Hasegawa and Duffull (2018)	pOBL	Ca, PO_4	Denosumab	1. Reduces Peterson and Riggs (2010) to a simple model<br>2. Reduced model captures long-term results
Pastrama et al. (2018)*	pOBL	-	-	1. Creates a model that considers the size and shape of BMU channel<br>2. Model has different concentration equations than Lemaire et al. (2004)<br>3. Explores different mechanical factors

Table S2 continued from previous page

Model	Cells	Signaling Mechanisms	Treatments	Motivation/Insights
Ji et al. (2019)	pOBL	-	Intracrine signaling	1. Develops a model that includes RANK<br>2. Investigates how OCLs respond to RANK activation
Lemaire and Cox (2019)	pOBL	SCL,<br>Wnt (assumed constant)	Denosumab, PTH, romosozumab	1. Focuses on the dynamics of cells with PTH and antibody treatments<br>2. Shows two coexisting pathways for PTH regulation
Martin et al. (2019)*	pOBL, OCY	NO, SCL,<br>Wnt (assumed constant)	-	1. Focuses on the biochemical response of a biomechanical stimulus<br>2. Model includes many commonly overlooked aspects
Martínez-Reina and Pivonka (2019)*	pOBL	-	Denosumab	1. Focuses on the long-term effects of denosumab<br>2. Model depicts the mineralization process<br>3. Suggests that the response to denosumab cannot be captured without alterations to the mineralization process
Trichilo et al. (2019)	pOBL	Intracrine signaling	PTH	1. Shows the dual action of administered PTH<br>2. Considers separate populations of existing OBLs and derived OBLs<br>3. Suggests that bone modeling might also be affected by administered PTH
Zhang and Mager (2019)	pOBL, MM	DKK1	Bortezomib, dexamethasone	1. Explores the optimal relationship between bortezomib and dexamethasone treatment<br>2. Identifies a potential regimen of drugs that is effective for treating MM while also preventing skeletal side effects
Ashrafi et al. (2020)*	pOBL	-	-	1. Derives a mechano-chemo-biological model using Lemaire et al. (2004) and various biomechanical models<br>2. Presents a new mechanotransduction approach that connects mechanical stimulus to a BMU
Bahia et al. (2020)*	pOBL	-	Bisphosphonate	1. Creates a mechano-chemo-biological model that includes PKPD and mechanical strain information information<br>2. Model shows bone response to pharmacological and mechanical influences

Table S2 continued from previous page

Model	Cells	Signaling Mechanisms	Treatments	Motivation/Insights
Ji et al. (2020)	pOBL, PC	Endo180, IL-6, SLRPs, VCAM1	-	1. Couples bone cells with tumor cells to explore disease development feedback<br>2. Explores the effects of various levels of TGF-β on Endo180 expression
Lavaill et al. (2020)*	pOBL	Intracrine signaling	PTH	1. Develops a mechanistic model of PTH treatment that includes mechanical feedback<br>2. Simulations suggest that a combination of PTH and mechanical loading is most effective
Martin et al. (2020)*	pOBL, OCY	NO, SCL, Wnt (assumed constant)	Romosozumab	1. Extends Martin et al. (2019) to include romosozumab<br>2. Model indicates need for other treatment after discontinuation of romosozumab
Larcher and Scheiner (2021)*	pOBL	-	-	1. Reduces the number of parameters in Scheiner et al. (2013)<br>2. Develops a framework for analyzing and reducing complex bone models
**PDEs**				
Buenzli et al. (2011)	pOBL	-	-	1. Focuses on the emergence of a BMU<br>2. Provides spatial understanding of how signaling factors cause cells to activate and migrate
Buenzli et al. (2014)	pOBL	-	-	1. Extends Buenzli et al. (2011) to include the formation phase<br>2. Points to the initiation and termination of a BMU to be much faster than other phases
Lerebours et al. (2016)*	pOBL, pOCL	-	-	1. Focuses on site-specific dynamics of bone<br>2. Bone loss from osteoporosis or disuse is dependent on site-specific dynamics
Kameo et al. (2020)*	OCY	SCL, Sema3A	Anti-RANKL antibody, anti-SCL antibody, Sema3A, bisphosphonate	1. Develops a platform of cell and organ interactions for in silico experiments<br>2. Highlights the importance of considering dual function molecules
Calvo-Gallego et al. (2023)*	pOBL, pOCL, OCY	-	-	1. Develops a spatiotemporal simulation of normal bone remodeling by extending Martínez-Reina and Pivonka (2019)<br>2. Shows that TGF-β helps coordinate the cycle

Table S3: Mathematical models of bone remodeling that do not follow either the power law or mass action kinetics approaches. Almost all the models in the table include osteoblasts (OBL) and osteoclasts (OCL), so these are not explicitly listed in the table. The exceptions are Akchurin et al. (2008), which only includes OCL, and Nutini et al. (2021) and Taylor-King et al. (2020), which exclude OCL. * denotes that a model has a biomechanical aspect. ^{d} denotes that a model is a system of delay differential equations. Abbreviations: agent-based models (ABMs), autocrine and paracrine (A&P) signaling, bone-derived factors (BDF), basic/bone multicellular unit (BMU), calcitonin (CT), dickkopf-related protein 1 (DKK1), hematopoietic stem cells (HSC), macrophage colony-stimulating factor (MCSF), mesenchymal stem cells (MSC), ordinary differential equations (ODEs), osteocytes (OCY), osteoprotegerin (OPG), parathyroid hormone (PTH), parathyroid hormone-related protein (PTHrP), partial differential equations (PDEs), preosteoblasts (pOBL), preosteoclasts (pOCL), prostate cancer (PC), receptor activator of nuclear factor kappa-B ligand (RANKL), sclerostin (SCL), transforming growth factor beta (TGF-β), wingless-related integration site (Wnt).

Model	Cells	Signaling Mechanisms	Treatments	Motivation/Insights
**ODEs**				
Moroz et al. (2006)*	OCY	A&P	-	1. Redevelops the A&P signaling to be more physiological and includes OCYs<br>2. Inclusion of OCYs allows for the entire remodeling cycle to be considered
Moroz and Wimpenny (2007)*	OCY	A&P	-	1. Adjusts Moroz et al. (2006) to include Hill function signaling for OCYs<br>2. Points to Hill functions as the preferred way to capture the complexity of remodeling
Akchurin et al. (2008)	Monocytes	RANKL	-	1. Captures the dynamic conversion of OCLs to monocytes<br>2. Kinetically models the conversion using *in vito* data
Ji et al. (2012)	-	-	-	1. Develops a predator-prey model that represents bone remodeling<br>2. Explores the population relationship of OBL and OCL in diseases
Buenzli (2015)	OCY	-	-	1. Develops a model of OCY generation and burial<br>2. Points to OCY burial being related to the infilling of the bone and the number of OCYs present
Proctor and Gartland (2016)*	pOBL, pOCL, OCY, HSC, MSC	Intracrine signaling, MCSF, OPG, PTH, RANKL, TGF-β, Wnt	-	1. Develops a model that includes mechanical and circadian rhythm influences<br>2. Explores the impact of mechanical loading and the timing of medical interventions

Table S3 continued from previous page

Model	Cells	Signaling Mechanisms	Treatments	Motivation/Insights
Chaiya and Rattanakul (2017)[d]	-	CT, PTH	Estrogen	1. Focuses on the effects of different hormones on bone remodeling<br>2. Shows that bone remodeling is highly sensitive to estrogen levels
Javed et al. (2018)	pOBL, pOCL	OPG, RANKL, SCL	Denosumab, estrogen	1. Develops a model that includes major influencers of osteoporosis<br>2. Model corroborates experimental results that point to denosumab repressing OCLs
Javed et al. (2020)	-	CT, OPG, RANKL	CT	1. Develops a model to explore CT as a therapeutic<br>2. CT alone can not provide treatment for osteoporosis
Zhao and Zhang (2019)	-	Generic signaling, PTH, PTHrP	PTHrP	1. Develops a simplified model of PTH influence on bone remodeling<br>2. Points to the benefit of utilizing bioengineered scaffolds for treating osteoporosis
Nutini et al. (2021)*	OCY	DKK1, OPG, RANKL, SCL	-	1. Models the SCL action on bone remodeling<br>2. Explores actions of mechanical stimulus and SCL
Jorg et al. (2022)	pOBL, pOCL, OCY	Estrogen, Generic signaling, SCL	Bisphosphonates, denosumab, PTH, romosozumab	1. Utilizes clinical data to develop a model of osteoporosis<br>2. Captures the dynamics of several treatment methods
**ABMs & PDEs**				
van Oers et al. (2008)*	OCY	-	-	1. Focuses on the cavities made by a BMU<br>2. Shows how OCLs move towards areas of OCY death
Buenzli et al. (2012a)	-	-	-	1. Shows the movement of OCLs and the corresponding bone resorption<br>2. Predicts the shape of the developed cavity
Araujo et al. (2014)	pOBL, pOCL, MSC, PC	BDF, RANKL, TGF-β	Anti-RANKL antibody, bisphosphonates	1. Focuses on the interactions occurring in the PC bone microenvironment<br>2. Includes distinct phases of bone calcium degradation and solidification<br>3. Shows efficacy of bisphosphonates

Table S3 continued from previous page

Model	Cells	Signaling Mechanisms	Treatments	Motivation/Insights
Arias et al. (2018)	pOBL, pOCL, OCY	Generic signaling	-	1. Determines the simplest model that can replicate remodeling<br>2. Points to a connection between the depth of OCY damage and OCL lifespan<br>3. Shows that a BMU can respond to a variety of damage regions
Taylor-King et al. (2020)	pOBL, OCY	-	Zolendrate (bisphosphonate)	1. Develops a model to depict the interconnectedness of OCYs<br>2. Explores how OCY network changes with cancer and bisphosphonates

References

Akchurin, T., Aissiou, T., Kemeny, N., Prosk, E., Nigam, N., and Komarova, S. V. (2008). Complex dynamics of osteoclast formation and death in long-term cultures. *PLoS ONE* 3, e2104. doi:10.1371/journal.pone.0002104

Araujo, A., Cook, L. M., Lynch, C. C., and Basanta, D. (2014). An integrated computational model of the bone microenvironment in bone-metastatic prostate cancer. *Cancer Res.* 74, 2391–2401. doi:10.1158/0008-5472.CAN-13-2652

Arias, C. F., Herrero, M. A., Echeverri, L. F., Oleaga, G. E., and López, J. M. (2018). Bone remodeling: A tissue-level process emerging from cell-level molecular algorithms. *PLoS ONE* 13, e0204171. doi:10.1371/journal.pone.0204171

Ashrafi, M., Gubaua, J. E., Pereira, J. T., Gahlichi, F., and Doblaré, M. (2020). A mechano-chemo-biological model for bone remodeling with a new mechano-chemo-transduction approach. *Biomech. Model. Mechan.* 19, 2499–2523. doi:10.1007/s10237-020-01353-0

Ayati, B. P., Edwards, C. M., Webb, G. F., and Wikswo, J. P. (2010). A mathematical model of bone remodeling dynamics for normal bone cell populations and myeloma bone disease. *Biol. Direct* 5, 28. doi:10.1186/1745-6150-5-28

Bahia, M., Hecke, M., Mercuri, E., and Pinheiro, M. (2020). A bone remodeling model governed by cellular micromechanics and physiologically based pharmacokinetics. *J. Mech. Behav. Biomed. Mater.* 104, 103657. doi:10.1016/j.jmbbm.2020.103657

Baldonedo, J. G., Fernández, J. R., and Segade, A. (2021). Spatial extension of a bone remodeling dynamics model and its finite element analysis. *Int. J. Numer. Meth. Bio.* 37. doi:10.1002/cnm.3429

Berkhout, J., Stone, J., Verhamme, K., Danhof, M., and Post, T. (2016). Disease systems analysis of bone mineral density and bone turnover markers in response to alendronate, placebo, and washout in postmenopausal women. *CPT: Pharmacometrics Syst. Pharmacol.* 5, 656–664. doi:10.1002/psp4.12135

Berkhout, J., Stone, J., Verhamme, K., Stricker, B., Sturkenboom, M., Danhof, M., et al. (2015). Application of a systems pharmacology-based placebo population model to analyze long-term data of postmenopausal osteoporosis. *CPT: Pharmacometrics Syst. Pharmacol.* 4, 516–526. doi:10.1002/psp4.12006

Buenzli, P. R. (2015). Osteocytes as a record of bone formation dynamics: A mathematical model of osteocyte generation in bone matrix. *J. Theor. Biol.* 364, 418–427. doi:10.1016/j.jtbi.2014.09.028

Buenzli, P. R., Jeon, J., Pivonka, P., Smith, D. W., and Cummings, P. T. (2012a). Investigation of bone resorption within a cortical basic multicellular unit using a lattice-based computational model. *Bone* 50, 378–389. doi:10.1016/j.bone.2011.10.021

Buenzli, P. R., Pivonka, P., Gardiner, B. S., and Smith, D. W. (2012b). Modelling the anabolic response of bone using a cell population model. *J. Theor. Biol.* 307, 42–52. doi:10.1016/j.jtbi.2012.04.019

Buenzli, P. R., Pivonka, P., and Smith, D. W. (2011). Spatio-temporal structure of cell distribution in cortical bone multicellular units: A mathematical model. *Bone* 48, 918–926. doi:10.1016/j.bone.2010.12.009

Buenzli, P. R., Pivonka, P., and Smith, D. W. (2014). Bone refilling in cortical basic multicellular units:

Insights into tetracycline double labelling from a computational model. *Biomech. Model. Mechan.* 13, 185–203. doi:10.1007/s10237-013-0495-y

Calvo-Gallego, J. L., Manchado-Morales, P., Pivonka, P., and Martínez-Reina, J. (2023). Spatio-temporal simulations of bone remodelling using a bone cell population model based on cell availability. *Front. Bioeng. Biotechnol.* 11, 1060158. doi:10.3389/fbioe.2023.1060158

Camacho, A. and Jerez, S. (2019). Bone metastasis treatment modeling via optimal control. *J. Math. Biol.* 78, 497–526. doi:10.1007/s00285-018-1281-3

Camacho, A. and Jerez, S. (2021). Nonlinear modeling and control strategies for bone diseases based on TGFβ and Wnt factors. *Commun. Nonlinear Sci. Numer. Simul.* 100, 105842. doi:10.1016/j.cnsns.2021.105842

Chaiya, I. and Rattanakul, C. (2017). An impulsive mathematical model of bone formation and resorption: Effects of parathyroid hormone, calcitonin and impulsive estrogen supplement. *Adv. Difference Equ.* 2017, 153. doi:10.1186/s13662-017-1206-2

Chen-Charpentier, B. M. and Diakite, I. (2016). A mathematical model of bone remodeling with delays. *J. Comput. Appl. Math.* 291, 76–84. doi:10.1016/j.cam.2014.11.025

Coelho, R. M., Lemos, J. M., Alho, I., Valério, D., Ferreira, A. R., Costa, L., et al. (2016). Dynamic modeling of bone metastasis, microenvironment and therapy: Integrating parathyroid hormone (PTH) effect, anti-resorptive and anti-cancer therapy. *J. Theor. Biol.* 391, 1–12. doi:10.1016/j.jtbi.2015.11.024

Cook, C. V., Islam, M. A., Smith, B. J., and Ford Versypt, A. N. (2022). Mathematical modeling of the effects of Wnt-10b on bone metabolism. *AIChE J.* 68, e17809. doi:10.1002/aic.17809

Eudy, R. J., Gastonguay, M. R., Baron, K. T., and Riggs, M. M. (2015). Connecting the dots: Linking osteocyte activity and therapeutic modulation of sclerostin by extending a multiscale systems model. *CPT: Pharmacometrics Syst. Pharmacol.* 4, 527–536. doi:10.1002/psp4.12013

Farhat, A., Jiang, D., Cui, D., Keller, E. T., and Jackson, T. L. (2017). An integrative model of prostate cancer interaction with the bone microenvironment. *Math. Biosci.* 294, 1–14. doi:10.1016/j.mbs.2017.09.005

Garzón-Alvarado, D. A. (2012). A mathematical model for describing the metastasis of cancer in bone tissue. *Comput. Methods Biomech. Biomed. Engin.* 15, 333–346. doi:10.1080/10255842.2010.535522

Graham, J. M. and Ayati, B. P. (2012). Towards a new spatial representation of bone remodeling. *Math. Biosci. Eng.* 9, 281–295. doi:10.3934/mbe.2012.9.281

Graham, J. M., Ayati, B. P., Holstein, S. A., and Martin, J. A. (2013). The role of osteocytes in targeted bone remodeling: A mathematical model. *PLoS ONE* 8, e63884. doi:10.1371/journal.pone.0063884

Hasegawa, C. and Duffull, S. B. (2018). Automated scale reduction of nonlinear QSP models with an illustrative application to a bone biology system. *CPT: Pharmacometrics Syst. Pharmacol.* 7, 562–572. doi:10.1002/psp4.12324

Idrees, M. and Sohail, A. (2020). A computational framework and sensitivity analysis for the hormonal treatment of bone. *Clin. Biomech.* 73, 9–16. doi:10.1016/j.clinbiomech.2019.12.015

Idrees, M. and Sohail, A. (2023). Optimizing the dynamics of bone turnover with genetic algorithm. *Model. Earth Syst. Environ.* 9, 1937–1947. doi:10.1007/s40808-022-01606-0

Idrees, M., Sohail, A., and Javed, S. (2019). Forecasting the critical role of intermittent therapies for the control of bone resorption. *Clin. Biomech.* 68, 128–136. doi:10.1016/j.clinbiomech.2019.04.023

Islam, M. A., Cook, C. V., Smith, B. J., and Ford Versypt, A. N. (2021). Mathematical modeling of the gut–bone axis and implications of butyrate treatment on osteoimmunology. *Ind. Eng. Chem. Res.* 60, 17814–17825. doi:10.1021/acs.iecr.1c02949

Javed, S., Sohail, A., Asif, A., and Nutini, A. (2020). Biophysics and the nonlinear dynamics instigated by a special hormone. *Prog. Biophys. Mol. Biol.* 150, 62–66. doi:10.1016/j.pbiomolbio.2019.05.005

Javed, S., Sohail, A., and Nutini, A. (2018). Integrative modeling of drug therapy and the bone turnover. *Clin. Biomech.* 60, 141–148. doi:10.1016/j.clinbiomech.2018.10.019

Javed, S., Younas, M., Bhatti, M. Y., Sohail, A., and Sattar, A. (2019). Analytic approach to explore dynamical osteoporotic bone turnover. *Adv. Difference Equ.* 2019, 61. doi:10.1186/s13662-019-1986-7

Jerez, S. and Chen, B. (2015). Stability analysis of a Komarova type model for the interactions of osteoblast and osteoclast cells during bone remodeling. *Math. Biosci.* 264, 29–37. doi:10.1016/j.mbs.2015.03.003

Jerez, S., Díaz-Infante, S., and Chen, B. (2018). Fluctuating periodic solutions and moment boundedness of a stochastic model for the bone remodeling process. *Math. Biosci.* 299, 153–164. doi:10.1016/j.mbs.2018.03.006

Ji, B., Chen, J., Zhen, C., Yang, Q., and Yu, N. (2020). Mathematical modelling of the role of Endo180 network in the development of metastatic bone disease in prostate cancer. *Comput. Biol. Med.* 117, 103619. doi:10.1016/j.compbiomed.2020.103619

Ji, B., Genever, P. G., Patton, R. J., and Fagan, M. J. (2014). Mathematical modelling of the pathogenesis of multiple myeloma-induced bone disease. *Int. J. Numer. Methods Biomed. Eng.* 30, 1085–1102. doi:10.1002/cnm.2645

Ji, B., Genever, P. G., Patton, R. J., Putra, D., and Fagan, M. J. (2012). A novel mathematical model of bone remodelling cycles for trabecular bone at the cellular level. *Biomech. Model. Mechanobiol.* 11, 973–982. doi:10.1007/s10237-011-0366-3

Ji, B., Zhang, Y., Zhen, C., Fagan, M. J., and Yang, Q. (2019). Mathematical modelling of bone remodelling cycles including the NFκB signalling pathway. *Comput. Biol. Med.* 107, 257–264. doi:10.1016/j.compbiomed.2019.03.003

Jorg, D. J., Fuertinger, D. H., Cherif, A., Bushinsky, D. A., Mermelstein, A., Raimann, J. G., et al. (2022). Modeling osteoporosis to design and optimize pharmacological therapies comprising multiple drug types. *eLife* 11, e76228. doi:10.7554/elife.76228

Kameo, Y., Miya, Y., Hayashi, M., Nakashima, T., and Adachi, T. (2020). In silico experiments of bone remodeling explore metabolic diseases and their drug treatment. *Sci. Adv.* 6, eaax0938. doi:10.1126/sciadv.aax0938

Komarova, S. V. (2005). Mathematical model of paracrine interactions between osteoclasts and osteoblasts predicts anabolic action of parathyroid hormone on bone. *Endocrinology* 146, 3589–3595. doi:10.1210/en.2004-1642

Komarova, S. V., Smith, R. J., Dixon, S. J., Sims, S. M., and Wahl, L. M. (2003). Mathematical model predicts a critical role for osteoclast autocrine regulation in the control of bone remodeling. *Bone* 33, 206–215. doi:10.1016/s8756-3282(03)00157-1

Kroll, M. (2000). Parathyroid hormone temporal effects on bone formation and resorption. *Bull. Math. Biol.* 62, 163–188. doi:10.1006/bulm.1999.0146

Larcher, I. and Scheiner, S. (2021). Parameter reduction, sensitivity studies, and correlation analyses applied to a mechanobiologically regulated bone cell population model of the bone metabolism. *Comput. Biol. Med.* 136, 104717. doi:10.1016/j.compbiomed.2021.104717

Lavaill, M., Trichilo, S., Scheiner, S., Forwood, M. R., Cooper, D. M. L., and Pivonka, P. (2020). Study of the combined effects of PTH treatment and mechanical loading in postmenopausal osteoporosis using a new mechanistic PK-PD model. *Biomech. Model. Mechanobiol.* 19, 1765–1780. doi:10.1007/s10237-020-01307-6

Lee, W.-H. and Okos, M. R. (2016). Model-based analysis of IGF-1 effect on osteoblast and osteoclast regulation in bone turnover. *J. Biol. Systems* 24, 63–89. doi:10.1142/s0218339016500042

Lemaire, V. and Cox, D. R. (2019). Dynamics of bone cell interactions and differential responses to PTH and antibody-based therapies. *Bull. Math. Biol.* 81, 3575–3622. doi:10.1007/s11538-018-0533-0

Lemaire, V., Tobin, F. L., Greller, L. D., Cho, C. R., and Suva, L. J. (2004). Modeling the interactions between osteoblast and osteoclast activities in bone remodeling. *J. Theor. Biol.* 229, 293–309. doi:10.1016/j.jtbi.2004.03.023

Lerebours, C., Buenzli, P. R., Scheiner, S., and Pivonka, P. (2016). A multiscale mechanobiological model of bone remodelling predicts site-specific bone loss in the femur during osteoporosis and mechanical disuse. *Biomech. Model. Mechanobiol.* 15, 43–67. doi:10.1007/s10237-015-0705-x

Liò, P., Paoletti, N., Moni, M. A., Atwell, K., Merelli, E., and Viceconti, M. (2012). Modelling osteomyelitis. *BMC Bioinf.* 13, S12. doi:10.1186/1471-2105-13-s14-s12

Marathe, A., Peterson, M. C., and Mager, D. E. (2008). Integrated cellular bone homeostasis model for denosumab pharmacodynamics in multiple myeloma patients. *J. Pharmacol. Exp. Ther.* 326, 555–562. doi:10.1124/jpet.108.137703

Marathe, D. D., Marathe, A., and Mager, D. E. (2011). Integrated model for denosumab and ibandronate pharmacodynamics in postmenopausal women. *Biopharm. Drug Dispos.* 32, 471–481. doi:10.1002/bdd.770

Martin, M., Sansalone, V., Cooper, D. M. L., Forwood, M. R., and Pivonka, P. (2019). Mechanobiological osteocyte feedback drives mechanostat regulation of bone in a multiscale computational model. *Biomech. Model. Mechanobiol.* 18, 1475–1496. doi:10.1007/s10237-019-01158-w

Martin, M., Sansalone, V., Cooper, D. M. L., Forwood, M. R., and Pivonka, P. (2020). Assessment of romosozumab efficacy in the treatment of postmenopausal osteoporosis: Results from a mechanistic PK-PD mechanostat model of bone remodeling. *Bone* 133, 115223. doi:10.1016/j.bone.2020.115223

Martínez-Reina, J. and Pivonka, P. (2019). Effects of long-term treatment of denosumab on bone mineral density: Insights from an in-silico model of bone mineralization. *Bone* 125, 87–95. doi:10.1016/j.bone.2019.04.022

Miranda, R., Vinga, S., and Valério, D. (2020). Studying bone remodelling and tumour growth for therapy predictive control. *Mathematics* 8, 679. doi:10.3390/math8050679

Moroz, A., Crane, M. C., Smith, G., and Wimpenny, D. I. (2006). Phenomenological model of bone remodeling cycle containing osteocyte regulation loop. *Biosystems* 84, 183–190. doi:10.1016/j.biosystems.2005.11.002

Moroz, A. and Wimpenny, D. I. (2007). Allosteric control model of bone remodelling containing periodical modes. *Biophys. Chem.* 127, 194–212. doi:10.1016/j.bpc.2007.02.001

Nutini, A., Sohail, A., and Farwa, S. (2021). Biomedical engineering of sclerostin action in the bone remodeling. *Biomed. Eng.: Appl. Basis Commun.* 33, 2150016. doi:10.4015/s1016237221500162

Pastrama, M.-I., Scheiner, S., Pivonka, P., and Hellmich, C. (2018). A mathematical multiscale model of bone remodeling, accounting for pore space-specific mechanosensation. *Bone* 107, 208–221. doi:10.1016/j.bone.2017.11.009

Peterson, M. C. and Riggs, M. M. (2010). A physiologically based mathematical model of integrated calcium homeostasis and bone remodeling. *Bone* 46, 49–63. doi:10.1016/j.bone.2009.08.053

Peterson, M. C. and Riggs, M. M. (2012). Predicting nonlinear changes in bone mineral density over time using a multiscale systems pharmacology model. *CPT: Pharmacometrics Syst. Pharmacol.* 1, e14. doi:10.1038/psp.2012.15

Peyroteo, M. M. A., Belinha, J., Dinis, L. M. J. S., and Natal Jorge, R. M. (2019). A new biological bone remodeling in silico model combined with advanced discretization methods. *Int. J. Numer. Methods Biomed. Eng.* 35, e3196. doi:10.1002/cnm.3196

Pivonka, P., Buenzli, P. R., Scheiner, S., Hellmich, C., and Dunstan, C. R. (2013). The influence of bone surface availability in bone remodelling–a mathematical model including coupled geometrical and biomechanical regulations of bone cells. *Eng. Struct.* 47, 134–147. doi:10.1016/j.engstruct.2012.09.006

Pivonka, P., Zimak, J., Smith, D. W., Gardiner, B. S., Dunstan, C. R., Sims, N. A., et al. (2008). Model structure and control of bone remodeling: A theoretical study. *Bone* 43, 249–263. doi:10.1016/j.bone.2008.03.025

Pivonka, P., Zimak, J., Smith, D. W., Gardiner, B. S., Dunstan, C. R., Sims, N. A., et al. (2010). Theoretical investigation of the role of the RANK–RANKL–OPG system in bone remodeling. *J. Theor. Biol.* 262, 306–316. doi:10.1016/j.jtbi.2009.09.021

Post, T. M., Schmidt, S., Peletier, L. A., de Greef, R., Kerbusch, T., and Danhof, M. (2013). Application of a mechanism-based disease systems model for osteoporosis to clinical data. *J. Pharmacokinet. Pharmacodyn.* 40, 143–156. doi:10.1007/s10928-012-9294-9

Proctor, C. J. and Gartland, A. (2016). Simulated interventions to ameliorate age-related bone loss indicate the importance of timing. *Front. Endocrinol.* 7, 61. doi:10.3389/fendo.2016.00061

Ross, D. S., Battista, C., Cabal, A., and Mehta, K. (2012). Dynamics of bone cell signaling and PTH treatments of osteoporosis. *Discrete Contin. Dyn. Syst. - B* 17, 2185–2200. doi:10.3934/dcdsb.2012.17.2185

Ross, D. S., Mehta, K., and Cabal, A. (2017). Mathematical model of bone remodeling captures the antiresorptive and anabolic actions of various therapies. *Bull. Math. Biol.* 79, 117–142. doi:10.1007/s11538-016-0229-2

Ryser, M. D., Komarova, S. V., and Nigam, N. (2010). The cellular dynamics of bone remodeling: A mathematical model. *SIAM J. Appl. Math.* 70, 1899–1921. doi:10.1137/090746094

Ryser, M. D. and Murgas, K. A. (2017). Bone remodeling as a spatial evolutionary game. *J. Theor. Biol.* 418, 16–26. doi:10.1016/j.jtbi.2017.01.021

Ryser, M. D., Nigam, N., and Komarova, S. V. (2009). Mathematical modeling of spatio-temporal dynamics of a single bone multicellular unit. *J. Bone Miner. Res.* 24, 860–870. doi:10.1359/jbmr.081229

Ryser, M. D., Qu, Y., and Komarova, S. V. (2012). Osteoprotegerin in bone metastases: Mathematical solution to the puzzle. *PLoS Comput. Biol.* 8, e1002703. doi:10.1371/journal.pcbi.1002703

Scheiner, S., Pivonka, P., and Hellmich, C. (2013). Coupling systems biology with multiscale mechanics, for computer simulations of bone remodeling. *Comput. Methods Appl. Mech. Eng.* 254, 181–196. doi:10.1016/j.cma.2012.10.015

Scheiner, S., Pivonka, P., Smith, D. W., Dunstan, C. R., and Hellmich, C. (2014). Mathematical modeling of postmenopausal osteoporosis and its treatment by the anti-catabolic drug denosumab. *Int. J. Numer. Methods Biomed. Eng.* 30, 1–27. doi:10.1002/cnm.2584

Schmidt, S., Post, T. M., Peletier, L. A., Boroujerdi, M. A., and Danhof, M. (2011). Coping with time scales in disease systems analysis: Application to bone remodeling. *J. Pharmacokinet. Pharmacodyn.* 38, 873–900. doi:10.1007/s10928-011-9224-2

Taylor-King, J. P., Buenzli, P. R., Chapman, S. J., Lynch, C. C., and Basanta, D. (2020). Modeling osteocyte network formation: Healthy and cancerous environments. *Front. Bioeng. Biotechnol.* 8, 757. doi:10.3389/fbioe.2020.00757

Trichilo, S., Scheiner, S., Forwood, M., Cooper, D. M. L., and Pivonka, P. (2019). Computational model of the dual action of PTH – application to a rat model of osteoporosis. *J. Theor. Biol.* 473, 67–79. doi:10.1016/j.jtbi.2019.04.020

van Oers, R. F. M., Ruimerman, R., Tanck, E., Hilbers, P. A. J., and Huiskes, R. (2008). A unified theory for osteonal and hemi-osteonal remodeling. *Bone* 42, 250–259. doi:10.1016/j.bone.2007.10.009

Wang, Y., Pivonka, P., Buenzli, P. R., Smith, D. W., and Dunstan, C. R. (2011). Computational modeling of interactions between multiple myeloma and the bone microenvironment. *PLoS ONE* 6, e27494. doi:10.1371/journal.pone.0027494

Wang, Y. and Qin, Q.-H. (2012). A theoretical study of bone remodelling under PEMF at cellular level. *Comput. Methods Biomech. Biomed. Engin.* 15, 885–897. doi:10.1080/10255842.2011.565752

Zhang, L. and Mager, D. E. (2019). Systems modeling of bortezomib and dexamethasone combinatorial effects on bone homeostasis in multiple myeloma patients. *J. Pharm. Sci.* 108, 732–740. doi:10.1016/j.xphs.2018.11.024

Zhao, Y. and Zhang, G. (2019). A computational study of the dual effect of intermittent and continuous administration of parathyroid hormone on bone remodeling. *Acta Biomater.* 93, 200–209. doi:10.1016/j.actbio.2019.04.007
